# From cartilage degeneration to pain sensitization: a multi-tissue mitochondrial dysfunction network in osteoarthritis

**DOI:** 10.3389/fcell.2026.1914283

**Published:** 2026-10-23

**Authors:** Kanghua Yang, Lin Li, Hui Yu

**Affiliations:** 1 Ganzhou Hospital-Nanfang Hospital, Southern Medical University (Ganzhou People’s Hospital), Ganzhou, Jiangxi Province, China; 2 The First Affiliated Hospital, Jiangxi Medical College,Nanchang University, Nanchang, Jiangxi Province, China

**Keywords:** chondrocyte senescence, mitochondrial dysfunction, mitochondria-targeted therapy, neuroimmune, osteoarthritis, pain sensitization, regulated cell death

## Abstract

Osteoarthritis (OA) is one of the most common degenerative joint diseases, and its clinical burden arises not only from degenerative changes and synovial inflammation but is also closely associated with chronic pain, functional impairment, and reduced quality of life. In patients with OA, imaging-detected structural damage and pain severity are not always consistent, suggesting that symptom persistence may also be driven by mechanisms such as metabolic disturbance, inflammatory amplification, and neural sensitization. Existing evidence indicates that mitochondrial abnormalities can be observed in cartilage, synovium, subchondral bone, and sensory nerve-related compartments, but the strength of evidence and the causal hierarchy differ across compartments. In chondrocytes, disruption of mitochondrial homeostasis is often accompanied by imbalanced energy metabolism, oxidative stress, impaired quality control, and mtDNA damage, and is associated with cell aging, regulated cell death, and matrix degeneration. Mitochondrial stress in other joint and neural compartments may participate in local inflammation, abnormal bone remodeling, and peripheral and central pain sensitization. Mitochondria-derived DAMPs, and inflammatory mediators may mediate cross-compartment interactions, but current evidence is insufficient to establish a continuous causal chain from cartilage degeneration unidirectionally to pain sensitization. Therefore, mitochondria in OA are more appropriately regarded as a pathological network shared across multiple tissue compartments and potentially interacting with one another. Building on this perspective, this article takes pain sensitization as an important dimension for understanding structural damage and symptoms in OA, and uses the “metabolism-senescence-pain axis” as a hypothesis-generating framework to review the role of mitochondrial dysfunction in OA structural degeneration, cell aging, regulated cell death, and pain sensitization, with emphasis on distinguishing evidence across cartilage, synovium, subchondral bone, sensory neurons, and central nervous system-related compartments, and to discuss potential cross-compartment interactions mediated by DAMPs, and neuroimmune signaling. Furthermore, by integrating human, animal, *in vitro*, and translational studies, the existing evidence is stratified, and the progress and translational barriers of mitochondria-directed and related therapeutic strategies are summarized. Finally, this article proposes future directions such as cell type-specific delivery, mitochondrial biomarkers, candidate pain mechanism phenotypes, and composite “mechanism-symptom-structure” endpoints, thereby offering a foundation for dissecting mechanistic heterogeneity, patient stratification, and precision intervention in OA.

## 1 Introduction

Osteoarthritis (OA) is one of the most common chronic joint diseases worldwide, and its pathological changes involve multiple levels, including articular cartilage degeneration, synovial inflammation, subchondral bone remodeling, and functional abnormalities of periarticular tissues (Yu et al., 2026; Arumugam et al., 2024). The traditional view often regards OA as a degenerative disease dominated by cartilage wear and structural degeneration, but a growing number of studies have shown that OA is not a simple mechanical injury process, but a complex disease driven by multiple pathological processes such as mechanical stress, metabolic disorders, low-grade inflammation, cellular senescence, and neuroimmune abnormalities (Guan and Mei, 2026; Liu et al., 2026). Clinically, patients with OA not only present with joint space narrowing, osteophyte formation, and cartilage destruction, but also often have chronic pain, limited activity, and reduced quality of life (Kim et al., 2024; Zhou et al., 2026). At present, OA treatment still mainly relies on analgesia, anti-inflammation, functional rehabilitation, and end-stage arthroplasty, and disease-modifying osteoarthritis drugs (DMOADs) that can effectively delay or reverse disease progression are still lacking (Brandt et al., 2025). Therefore, elucidating the pathological basis for the discordance between structural degeneration and chronic pain is critical for exploring the mechanistic heterogeneity of OA and for mechanism-oriented patient stratification and treatment strategies.

It is worth noting that the degree of imaging structural damage and the severity of pain in patients with OA are not always linearly consistent. Some patients do not have prominent pain symptoms even when obvious cartilage degeneration and osteophyte formation are present; while other patients may experience persistent and intractable pain with only mild to moderate structural changes. This phenomenon of “structure-symptom mismatch” suggests that OA pain cannot be fully explained by cartilage wear or joint structural destruction, and may also involve mechanisms such as synovial inflammation, subchondral bone abnormalities, sensitization of peripheral nociceptors, and abnormal central pain regulation (Khotib et al., 2020; Sharma and Sharma, 2026). Previous studies have explained OA structural degeneration from the perspectives of matrix degradation, inflammation, mechanical load, and cell death, but a mechanistic framework that can simultaneously explain tissue injury and pain sensitization and integrate metabolic, inflammatory, and neural signals is still insufficient (Xie et al., 2021; Peng et al., 2024). Therefore, identifying the shared but not completely overlapping pathological mechanisms in structural lesions and pain persistence, as well as the interactions among different tissue compartments, has become an important direction in OA research.

In recent years, increasing evidence suggests that mitochondrial dysfunction is not limited to chondrocytes, but may simultaneously exist in multiple pathological compartments such as cartilage, synovium, subchondral bone, and the sensory nervous system. This dysfunction is associated with OA structural degeneration and pain sensitization, and may actively contribute to these pathological processes. As a core hub of energy metabolism, redox homeostasis, calcium homeostasis, and cell fate regulation, mitochondria play a key role in maintaining chondrocyte homeostasis (Xin R. et al., 2022). Under OA conditions, factors such as abnormal mechanical load, inflammatory stimulation, and obesity-related lipotoxicity can disrupt mitochondrial homeostasis, causing energy metabolic imbalance, enhanced oxidative stress, impaired mitochondrial quality control, and mitochondrial DNA (mtDNA) damage (Wang S. et al., 2026; Xu X. et al., 2025). These changes may further contribute to chondrocyte senescence, the senescence-associated secretory phenotype (SASP), and various forms of regulated cell death (RCD), and are associated with matrix degradation and joint inflammation (Liu et al., 2025; Afzal et al., 2025). At the same time, mitochondria-derived reactive oxygen species, mtDNA, and other damage-associated molecular patterns (DAMPs) may also target the synovium, subchondral bone, sensory neurons, and glial cells, thereby amplifying neuroimmune signals and peripheral/central pain sensitization (Olatona et al., 2025; Tian et al., 2025). Therefore, mitochondrial abnormalities are more appropriately understood as shared pathological features and a potential interaction network spanning joint and neural compartments and coupled by metabolic stress, inflammation, and neuroimmune signals, rather than a linear “bridge” from structural degeneration unidirectionally leading to pain sensitization.

Although existing reviews have synthesized mitochondrial dysfunction, cartilage degeneration, and altered cell fate in OA, pain sensitization is usually not integrated as a core dimension in parallel with structural degeneration. Therefore, guided by the clinical problem of “mismatch between structural damage and pain severity,” this article incorporates pain sensitization into the mitochondrial pathological framework and divides the relevant evidence into three interrelated but non-equivalent levels. These include evidence that mitochondrial dysfunction contributes to structural joint pathology (e.g., cartilage degeneration, synovial inflammation, and subchondral bone remodeling); evidence that mitochondrial abnormalities in local joint and nervous system compartments contribute to pain generation and sensitization; and evidence that different tissue compartments form mechanistic links between structural lesions and pain sensitization through DAMPs, inflammatory mediators, and neuroimmune signals. The evidence for the first two aspects is comparatively robust, while research directly supporting cross-compartment causal links remains scarce. Therefore, when discussing cross-compartment links, this article will clearly distinguish the tissue, cell type, and experimental model from which the evidence is derived, avoiding direct extrapolation of mechanisms in chondrocytes to the synovium, subchondral bone, dorsal root ganglia (DRG)/sensory neurons, or the central nervous system.

Based on this, this article constructs an integrative framework around the “metabolism-senescence-pain axis.” First, it outlines chondrocyte mitochondrial metabolic homeostasis and quality control. Subsequently, it analyzes how respiratory chain damage, mtROS accumulation, dynamic imbalance, insufficient mitophagy, and mtDNA damage in OA contribute to cell senescence and RCD. On this basis, it further focuses on the possible mechanistic links between these abnormalities and peripheral and central pain sensitization in the synovium, subchondral bone, and nervous system. Finally, this article evaluates the existing evidence and translational barriers for mitochondrial-targeted therapy, and proposes future research directions such as cell type-specific delivery, validation of candidate pain mechanism phenotypes, mitochondria-related biomarkers, and mechanism-symptom-structure composite endpoints. In this article, the “metabolism-senescence-pain axis” serves as a conceptual framework for integrating different pathological compartments and disease phenotypes, and does not presuppose a fixed unidirectional causal order from cartilage degeneration to pain sensitization. This article aims to explore how cross-tissue mitochondrial abnormalities and their interactions jointly contribute to the formation of OA structural and pain phenotypes, and to provide a theoretical basis for future work on exploring mechanistic heterogeneity, stratifying candidate patients, and enabling precision intervention.

## 2 Mitochondrial metabolic homeostasis in normal chondrocytes

Articular cartilage is a specialized tissue that is avascular, lacks lymphatic vessels, and is not innervated. Its nutrient supply mainly depends on diffusion from synovial fluid, so chondrocytes are chronically exposed to a hypoxic and nutrient-poor microenvironment (Bačenková et al., 2023). Under physiological conditions, the oxygen partial pressure in cartilage tissue is approximately 2%–7%, a feature that drives chondrocytes to rely primarily on glycolysis for energy supply rather than depending entirely on mitochondrial oxidative phosphorylation (OXPHOS) (Tanaka et al., 2022; Pattappa et al., 2023). Studies have shown that even under aerobic conditions, chondrocytes tend to adopt a metabolic mode similar to aerobic glycolysis, that is, rapidly producing ATP by enhancing glucose uptake and lactate production (Stegen et al., 2022). This metabolic pattern helps chondrocytes adapt to the hypoxic environment and reduces the risk of oxidative stress caused by excessive activity of the mitochondrial respiratory chain. However, mitochondria are not marginal organelles in chondrocytes. Although glycolysis contributes most of the ATP source in chondrocytes, mitochondria still provide part of the energy through OXPHOS and participate in the tricarboxylic acid cycle, generation of reducing equivalents, supply of biosynthetic precursors, and regulation of cell fate (Bao et al., 2022; Zheng L. et al., 2021). Therefore, the metabolic homeostasis of normal chondrocytes is not simply “glycolysis-dominated,” but depends on dynamic coordination between glycolysis and mitochondrial function.

In addition to energy metabolism, mitochondria also maintain chondrocyte homeostasis by regulating redox balance, calcium homeostasis, and metabolic sensing pathways. Under physiological conditions, the mitochondrial electron transport chain produces a small amount of reactive oxygen species (ROS), which can act as signaling molecules participating in cellular adaptive responses; however, their production and clearance must remain balanced to avoid oxidative stress damage (Chen et al., 2024). The stability of the mitochondrial membrane potential (ΔΨm) is crucial for maintaining ATP synthesis, ROS homeostasis, and cell survival (Tang et al., 2026). At the same time, as an important intracellular calcium buffering system, mitochondria can regulate cytosolic calcium fluctuations through the mitochondrial calcium uniporter and voltage-dependent anion channel (VDAC), thereby affecting cell metabolism, matrix synthesis, and stress responses (Zhang L. et al., 2022; Shehwar et al., 2025).

To avoid classifying all signals related to mitochondrial function as “mitochondrial targets,” this article distinguishes between direct mitochondrial targets/processes and mitochondria-related pathways. The former mainly includes molecules and processes directly localized to mitochondria or directly regulating their structure and function, such as the respiratory chain/OXPHOS, ΔΨm, mitochondrial permeability transition pore (mPTP)/VDAC-mediated membrane permeability, mitochondrial dynamics, PINK1/Parkin-related mitophagy, mtDNA, and SIRT3; the latter includes SIRT1/PGC-1α, AMPK/mTOR, NF-κB/MAPK, cGAS-STING, NLRP3, as well as SASP and related cell death pathways. These pathways can be affected by mitochondrial status or can conversely regulate mitochondrial function, but they are not equivalent to mitochondria-specific targets (Chen R. et al., 2025; Xian et al., 2022; Lan et al., 2026). This difference is especially pronounced in metabolic sensing networks. SIRT3 is mainly localized in the mitochondrial matrix and can directly regulate redox homeostasis by deacetylating mitochondrial proteins such as SOD2; in contrast, SIRT1 is mainly located in the nucleus and cytoplasm and indirectly affects mitochondrial biogenesis and antioxidant defense through factors such as PGC-1α and FOXO (Sun et al., 2022; Zeng and Chen, 2022); AMPK/mTOR belongs to cellular energy and nutrient sensing pathways and can regulate autophagy, mitophagy, and metabolic adaptation according to energy status, while PGC-1α mainly acts as a nuclear transcriptional coactivator to regulate mitochondrial biogenesis (Ruan et al., 2024). Therefore, SIRT1, AMPK/mTOR, and PGC-1α are more appropriately classified as mitochondria-related metabolic regulatory pathways rather than direct mitochondrial targets.

Mitochondrial quality control (MQC) is a core system for maintaining mitochondrial homeostasis in chondrocytes and primarily encompasses three mutually coordinated processes: mitochondrial dynamics, mitophagy, and mitochondrial biogenesis (Wu et al., 2026). Mitochondrial dynamics maintains the morphological and functional integrity of the mitochondrial network through fission and fusion, in which fusion is mainly mediated by MFN1/2 and OPA1 and helps dilute damaged mitochondrial components and achieve functional complementation; fission is mainly regulated by DRP1 and separates damaged mitochondrial fragments from the healthy mitochondrial network, thereby creating conditions for subsequent clearance (Vezza et al., 2022; Singh and Kumar, 2023). Mitophagy selectively clears functionally damaged mitochondria through classical pathways such as PINK1/Parkin, preventing their accumulation in cells and the sustained release of ROS or pro-inflammatory signals (Sauvé and Gehring, 2025; Zhang T. et al., 2022). Meanwhile, PGC-1α/NRF1/TFAM coordinates mitochondrial biogenesis by promoting the expression of mitochondria-related genes, maintaining mtDNA, and facilitating the formation of new mitochondria to replenish the healthy mitochondrial pool (Kim and Hwang, 2020; Sun X. et al., 2023). Among these, PGC-1α and NRF1 are primarily responsible for nuclear transcriptional regulation, whereas TFAM directly participates in mtDNA maintenance inside mitochondria, thereby reflecting distinct levels of regulation between mitochondria-related control and direct mitochondrial effects (Tian et al., 2023).

Overall, chondrocyte mitochondrial homeostasis depends on coordination among energy metabolism, membrane function, dynamics, selective clearance, and biogenesis. These direct mitochondrial processes are in turn regulated by cellular metabolic and stress signaling networks. When the balance between the two is impaired, mitochondrial damage, oxidative stress, and metabolic abnormalities can gradually accumulate, providing an important basis for OA-related chondrocyte senescence, regulated cell death, and other pathological changes (Figure 1).

**FIGURE 1:**
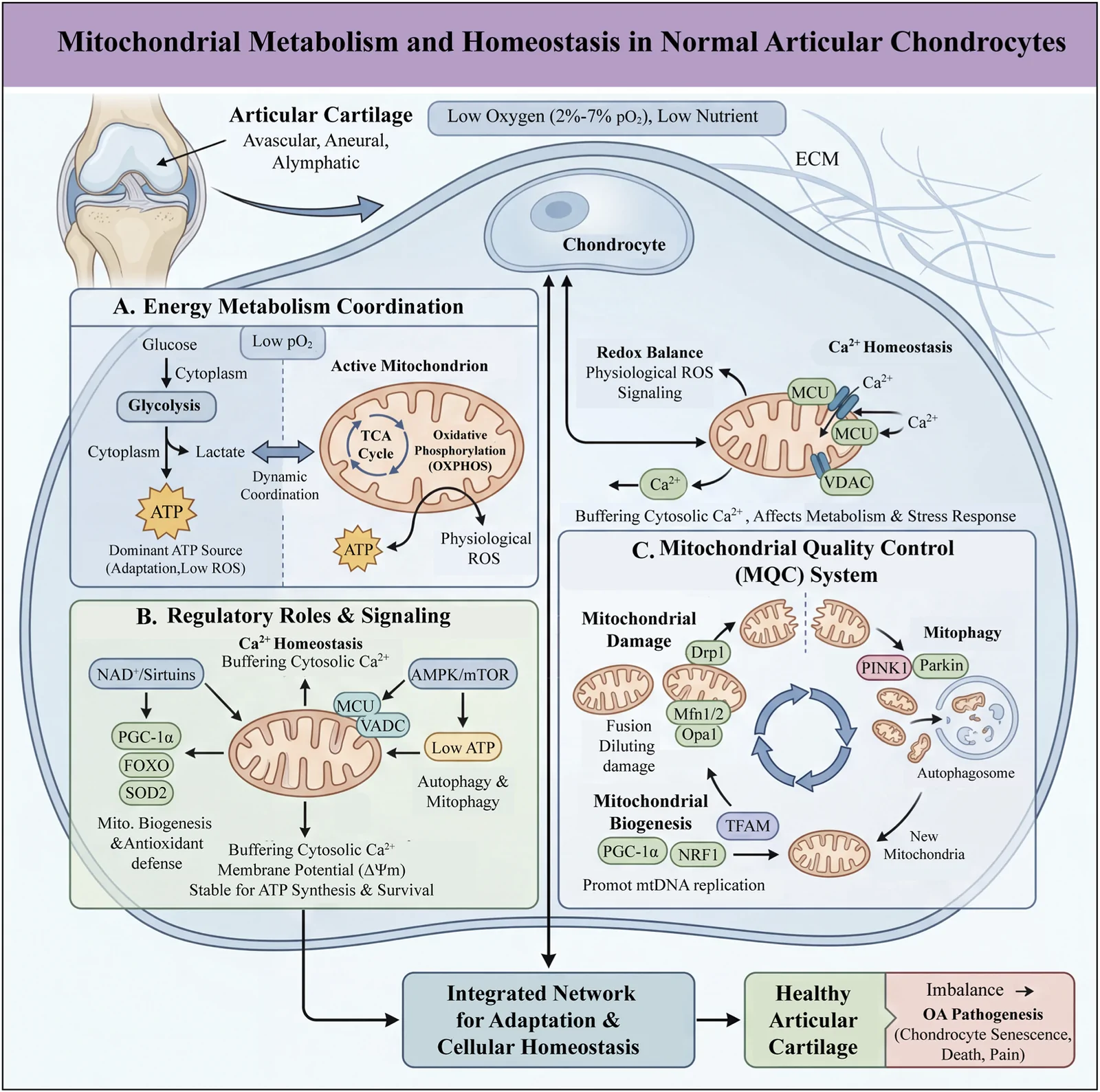
Mechanisms of mitochondrial metabolism and homeostasis maintenance in normal articular chondrocytes. Articular cartilage exists in a special hypoxic, nutrient-poor, avascular, aneural, and alymphatic microenvironment, and chondrocytes rely on dynamic coordination between glycolysis and mitochondrial oxidative metabolism to maintain energy supply and low-level ROS signaling. **(A)** Coordination of energy metabolism: glycolysis is the main source of ATP in chondrocytes, and mitochondria participate in energy supplementation and physiological ROS signaling regulation through the TCA cycle and oxidative phosphorylation. **(B)** Regulatory effects and signal transduction: mitochondria regulate antioxidant defense, autophagy/mitophagy, and cell survival through NAD^+^/Sirtuins, PGC-1α, AMPK/mTOR, Ca^2+^ homeostasis, and membrane potential. **(C)** Mitochondrial quality control system: mitochondrial fission/fusion, PINK1/Parkin-mediated mitophagy, and PGC-1α/NRF1/TFAM-mediated mitochondrial biogenesis jointly maintain mitochondrial renewal and functional homeostasis. These processes together support chondrocyte homeostasis, and their imbalance may provide a pathological basis for OA-related chondrocyte senescence, cell death, and disruption of matrix homeostasis.

## 3 Mitochondrial dysfunction across multiple tissue compartments in OA: shared mechanisms and potential cross-tissue interactions

Mitochondrial abnormalities in OA involve interconnected processes including respiratory chain damage, mtROS accumulation, dynamic imbalance, insufficient mitophagy, and mtDNA damage. Respiratory chain impairment can reduce ATP production and membrane potential while increasing electron leakage and mtROS; persistent oxidative stress further damages mitochondrial proteins, membrane lipids, and mtDNA, and disrupts dynamics and quality control (Tian et al., 2023; Cheung et al., 2024). Once damaged mitochondria accumulate, mtDNA and other mitochondrial DAMPs can further activate sterile inflammation and stress pathways, coupling mitochondrial damage and cellular inflammatory responses bidirectionally (Barrera et al., 2021). According to the aforementioned conceptual framework, this section treats the respiratory chain/OXPHOS, ΔΨm, mPTP/VDAC, mitochondrial dynamics, PINK1/Parkin-related mitophagy, and mtDNA as direct mitochondrial processes, while TLR9, cGAS-STING, NLRP3, NF-κB, and MAPK are primarily discussed as downstream inflammatory/stress pathways responsive to mitochondrial DAMPs or oxidative stress. Unless otherwise specified, the OA-specific mechanistic evidence regarding the above mitochondrial abnormalities in this section is mainly derived from chondrocytes or cartilage tissue; evidence from synovium, subchondral bone, immune cells, and the nervous system will be separately noted at the corresponding positions and should not be directly extrapolated from chondrocyte studies. Similar mitochondrial abnormalities are also observed in other joint and neural compartments, but these changes may occur independently or produce cross-compartment interactions through DAMPs, cytokines, and metabolic signals. It should be emphasized that the appearance of similar mitochondrial phenotypes in different compartments does not imply shared cellular origin, temporal sequence, or causal role, nor should it be inferred that the same mitochondrial abnormality simultaneously drives structural OA progression and pain sensitization. The current evidence is insufficient to prove that chondrocyte mitochondrial dysfunction represents a necessary mechanistic link between structural cartilage degeneration and pain sensitization. Therefore, the term “multi-compartment mitochondrial network” used in this article primarily describes shared or potentially interacting pathological features across different tissue compartments, rather than implying a continuous causal chain from cartilage to the pain system.

### 3.1 Respiratory chain dysfunction and energy crisis

In the pathological progression of OA, mitochondrial respiratory chain dysfunction is an important manifestation of chondrocyte energy metabolism disorder. Studies have shown that reduced activities of mitochondrial respiratory chain complex I and complex III in OA chondrocytes can lead to decreased electron transport efficiency, impaired OXPHOS, and reduced ATP production, accompanied by increased electron leakage and ROS production (Eitner et al., 2022; Zhong et al., 2022). Decreased ΔΨm further weakens ATP synthesis capacity and, together with ROS accumulation and Ca^2+^ homeostasis disturbance, can promote sustained opening of the mPTP. Excessive mPTP opening can lead to further collapse of ΔΨm, mitochondrial swelling, and ATP depletion, and promotes the release of mitochondrial contents and cell death (Valdeben et al., 2022; Rovini et al., 2021). Recent OA studies also suggest that abnormal mPTP opening can promote the entry of damaged mtDNA into the cytoplasm, subsequently activating cGAS-STING-related inflammatory responses, reflecting a mechanistic link between direct mitochondrial membrane dysfunction and downstream immune signaling (Liu J. et al., 2024). The VDAC, located in the mitochondrial outer membrane, serves as a critical regulator of transmembrane exchange of ATP/ADP, metabolites, and Ca^2+^. Under mitochondrial stress, abnormal VDAC function or oligomerization can alter outer membrane permeability and may promote the release of danger signals such as cytochrome c and mtDNA (Yan J. et al., 2020). Therefore, mPTP- and VDAC-related changes in membrane permeability constitute important amplification steps following respiratory chain functional decline and may participate in the interactions among ROS/Ca^2+^ imbalance, energy depletion, danger signal release, and cell death. However, the specific roles of VDAC in different OA joint cells and its functional coupling with mPTP still require further verification.

Decreased mitochondrial respiratory function is also manifested as reduced oxygen consumption rate and respiratory control ratio, suggesting impaired respiratory coupling efficiency in OA chondrocytes (Eitner et al., 2022). To compensate for energy deficiency, cells may increase glycolysis, but sustained metabolic reprogramming can cause lactate accumulation, intracellular acidification, and decreased matrix synthesis capacity, further disrupting extracellular matrix (ECM) homeostasis (Wu et al., 2023). Thus, respiratory chain damage not only causes ATP deficiency but also establishes a pathological basis for subsequent mtROS accumulation, mitochondrial dynamic abnormalities, and mitochondrial quality control failure.

### 3.2 mtROS accumulation and oxidative stress amplification

mtROS accumulation is an important amplifying signal through which respiratory chain damage extends to other mitochondrial abnormalities. Under normal conditions, the mitochondrial electron transport chain produces small amounts of ROS that serve as signaling molecules in cellular adaptive regulation; however, under OA pathological conditions, decreased respiratory chain complex activity, loss of ΔΨm, and increased electron leakage can lead to excessive mtROS production (Fernández-Moreno et al., 2022). Excessive mtROS can attack lipids, proteins, and nucleic acids, inducing lipid peroxidation, protein oxidative modification, and mtDNA damage, and further weakening respiratory chain function, forming a self-amplifying loop of “respiratory chain damage-mtROS accumulation-further mitochondrial damage” (Byrne et al., 2021; Banerjee et al., 2020).

In chondrocytes, mtROS can also affect ECM homeostasis. ROS can activate downstream inflammatory and stress pathways such as NF-κB and MAPK, upregulate catabolic molecules such as MMPs and ADAMTS, and promote degradation of type II collagen and aggrecan (Ansari et al., 2020); simultaneously, they can inhibit the expression of anabolic genes such as COL2A1 and ACAN (Liu et al., 2023). Persistent oxidative stress can also activate senescence-related pathways such as p53/p21 and p16^INK4a^, and promote SASP formation (Chen H. et al., 2023). Therefore, NF-κB, MAPK, and senescence-related pathways in this context mainly represent downstream cellular responses to mitochondrial oxidative stress, rather than direct mitochondrial targets.

In addition to directly damaging the mitochondrial matrix and inducing cellular senescence, mtROS can further disrupt mitochondrial dynamics and quality control. Persistent oxidative stress can promote DRP1-related mitochondrial fission, exacerbate mitochondrial network fragmentation, and increase the burden of damaged mitochondria that need to be cleared by mitophagy (Kim MJ. et al., 2022). Therefore, when subsequent dynamic remodeling and clearance capacity are insufficient, mtROS can further become an important amplifying factor maintaining the mitochondrial damage cycle, beyond being a consequence of respiratory chain damage.

### 3.3 Mitochondrial dynamics imbalance

Mitochondrial dynamics maintain the structural and functional integrity of the mitochondrial network through fusion, fission, and mitochondrial transport, and participate in the isolation, functional complementation, and subsequent clearance of damaged mitochondria (Awasthi et al., 2025). In OA chondrocytes, this balance is often disrupted, manifesting as enhanced fission and insufficient fusion. DRP1 is a core regulatory protein of mitochondrial fission, and inflammatory stimuli such as IL-1β can promote its activation or mitochondrial translocation, leading to fragmentation of the mitochondrial network (Shen et al., 2025; Ye et al., 2026). Excessive fission can reduce OXPHOS efficiency, decrease ATP production, and increase ROS release, thereby further aggravating metabolic stress and oxidative damage (Ansari et al., 2022). Conversely, MFN1/2 and OPA1 regulate outer and inner mitochondrial membrane fusion, respectively, and their functional decline can weaken mitochondrial content exchange, mtDNA complementation, and functional recovery (Moqbel et al., 2022; Madhu et al., 2025). Furthermore, mechanical and inflammatory signals can regulate MFN1/2 or DRP1 through upstream pathways such as YAP, TRPV4, and TBK1, thereby affecting mitochondrial network homeostasis (Cheng et al., 2026).

In addition to classical fusion/fission proteins, novel regulatory factors involved in mitochondrial transport and quality control, such as Miro1 (RHOT1), have also attracted attention. Miro1 is localized to the mitochondrial outer membrane and can regulate mitochondrial positioning, transport, and PINK1/Parkin-related quality control (Schwarz et al., 2021). Recent OA studies have shown that in the DMM model, osteocyte-specific Miro1 deletion can reduce mitochondrial transfer from osteocytes to chondrocytes, accompanied by attenuated cGAS-STING signaling, ameliorated cartilage degeneration and synovitis, and reduced mechanical hyperalgesia (Ma et al., 2025). This interventional study provides preliminary mechanistic evidence for mitochondrial transfer between the specific compartments of osteocytes and chondrocytes, and suggests that it may participate in local intercellular communication in OA. However, this result cannot be extrapolated to a universally existing mitochondrial transfer mechanism among cartilage, synovium, immune cells, and sensory nerves, nor can it prove that chondrocyte mitochondrial abnormalities directly drive pain sensitization through this process. Similarly, the simultaneous occurrence of structural improvement and improvement in pain-related behavior in that study cannot alone prove that both are mediated by the same mitochondrial abnormality or the same pathway. Its cell type specificity and role in different OA stages still require further verification.

Dynamic imbalance is closely coupled with subsequent mitophagy. Moderate fission helps isolate functionally impaired parts from the healthy mitochondrial network for subsequent selective clearance; however, persistent excessive fission, insufficient fusion, or abnormal transport can lead to the accumulation of large numbers of low-function mitochondrial fragments and interfere with their recognition and clearance (Wu et al., 2020; Li R. et al., 2020). Therefore, dynamic abnormalities can be driven by ROS and inflammation, and can in turn reduce respiratory efficiency, increase ROS and mtDNA damage, and further promote mitochondrial quality control failure.

### 3.4 Insufficient mitophagy and MQC failure

Mitophagy is a core component of MQC, responsible for selectively clearing damaged or functionally abnormal mitochondria (Marx et al., 2025). When the aforementioned respiratory chain damage, mtROS accumulation, and dynamic abnormalities persist, the prompt clearance of damaged mitochondria by mitophagy determines whether pathological stress is terminated or further amplified. The classical PINK1/Parkin pathway is the most commonly studied mitophagy pathway. When mitochondrial membrane potential decreases, PINK1 stabilizes on the mitochondrial outer membrane and recruits Parkin, initiating ubiquitination of outer membrane proteins and autophagosome encapsulation (Malpartida et al., 2021). However, in OA chondrocytes, PINK1 and Parkin expression or function are frequently inhibited, preventing damaged mitochondria from being effectively recognized and cleared (Li Q. et al., 2025).

In addition to the PINK1/Parkin pathway, receptor-mediated mitophagy involving BNIP3, NIX, and FUNDC1 also participates in chondrocyte adaptation to hypoxia and stress environments. Under OA conditions, these alternative pathways may also become dysregulated, leading to insufficient clearance of damaged mitochondria or imbalanced autophagic responses (Turkieh et al., 2022; Sun et al., 2021). It should be noted that mitophagy in OA has a dual role: moderate mitophagy helps clear damaged mitochondria, reduce mtROS, and maintain cellular homeostasis; whereas excessive or imbalanced autophagic responses may aggravate energy consumption and cellular damage (Fang et al., 2023; Hu et al., 2020). Therefore, when evaluating mitophagy in OA, attention should be paid to the complete autophagic flux process, and its protective or damaging role should not be judged based on a single autophagy marker alone.

When mitophagy is insufficient, mitochondria with impaired respiratory function, high ROS production, and mtDNA damage continuously accumulate, which can further increase mtROS and mtDNA release (Xu Y. et al., 2026; Kim JS. et al., 2022). Inflammatory factors can in turn inhibit autophagic flux, transforming transient mitochondrial stress into sustained quality control failure (Li XQ. et al., 2024). Therefore, insufficient mitophagy is not an independent endpoint, but an important link participating in the mutual amplification of dynamic imbalance, accumulation of damaged mitochondria, and downstream DAMPs release.

### 3.5 mtDNA damage, DAMP release, and sterile inflammation

mtDNA damage and its release as DAMPs are important links through which the aforementioned mitochondrial damage network contributes to sterile inflammation. Compared with nuclear DNA, mtDNA lacks histone protection, has relatively limited repair capacity, and is located near ROS production sites, making it more susceptible to oxidative damage (Yang C. et al., 2026; Cai et al., 2025). Under OA conditions, mtROS accumulation can induce mtDNA base oxidation, strand breaks, and copy number abnormalities; damaged mtDNA further enters the cytoplasm or extracellular environment and is recognized by pattern recognition receptors as DAMPs (Fan et al., 2025; Yan et al., 2023). Altered mitochondrial membrane permeability may be one important pathway for mtDNA release. In addition to mPTP-related disruption of membrane integrity, pores formed by VDAC oligomerization are also considered to promote the release of mtDNA fragments into the cytoplasm, thereby linking respiratory chain damage, ROS accumulation, and membrane permeability abnormalities to innate immune signaling (Kim et al., 2023).

Released mtDNA can amplify inflammatory responses through multiple innate immune pathways. Recognition of oxidized mtDNA by Toll-like receptor 9 (TLR9) promotes the expression of pro-inflammatory factors such as TNF-α, IL-6, and IL-1β through MyD88-NF-κB signaling (Ward et al., 2023; Ye et al., 2023). Cytoplasmic mtDNA can also activate the cGAS-STING pathway, inducing IRF3 phosphorylation and type I interferon-related responses (Yan J. et al., 2020; Jauhari et al., 2020). Meanwhile, mtDNA and mtROS can synergistically promote NLRP3 inflammasome activation, enhancing caspase-1-mediated maturation and release of IL-1β and IL-18 (Barrera et al., 2021; Liao et al., 2022). Importantly, TLR9, cGAS-STING, NLRP3, and their downstream NF-κB/IRF3 are not mitochondria-specific targets, but rather downstream innate immune/inflammatory pathways capable of sensing or responding to mitochondria-derived DAMPs and oxidative stress. Therefore, they are classified in this article as “mitochondria-related pathways” rather than direct mitochondrial mechanisms.

These inflammatory pathways can in turn increase oxidative stress, affect respiratory chain function, and influence mitochondrial quality control, thereby forming bidirectional feedback between mitochondrial damage and inflammation. The above intracellular links have been supported by many mechanistic experiments, but whether they cross different joint and neural compartments in the same manner remains uncertain. Mitochondrial DAMPs and related inflammatory mediators may affect synovium, subchondral bone, immune cells, and sensory nerve-related cells, and participate in cross-tissue pathological interactions (Oduro et al., 2022). However, based on current evidence, it is necessary to distinguish between “mitochondrial dysfunction existing in parallel in different compartments” and “mitochondrial abnormalities in one compartment causally driving pathological changes in another compartment.” In particular, there is currently insufficient evidence to prove that chondrocyte mitochondrial damage forms a continuous “cartilage degeneration–cross-tissue mitochondrial abnormality–pain sensitization” causal pathway via DAMPs or other signals. Therefore, respiratory chain impairment, oxidative stress, dynamic abnormalities, insufficient mitophagy, and mtDNA/DAMPs release in OA can be regarded as mitochondrial-related pathological features that repeatedly appear across multiple tissue compartments. The appearance of these similar phenotypes in different tissue compartments cannot prove that they share a common origin or serve the same pathological function; in particular, it cannot be inferred that the same mitochondrial abnormality is simultaneously responsible for both phenotypes merely because a certain mitochondrial pathway is separately associated with structural degeneration and pain. These abnormalities may occur independently of each other, or may form limited cross-compartment interactions through inflammatory, metabolic, and neuroimmune signals; at the current stage, it is more appropriate to understand them as a multi-compartment shared pathological network, rather than an established mechanistic chain in which chondrocyte mitochondrial dysfunction unidirectionally drives whole-joint pathology and pain sensitization (Figure 2).

**FIGURE 2:**
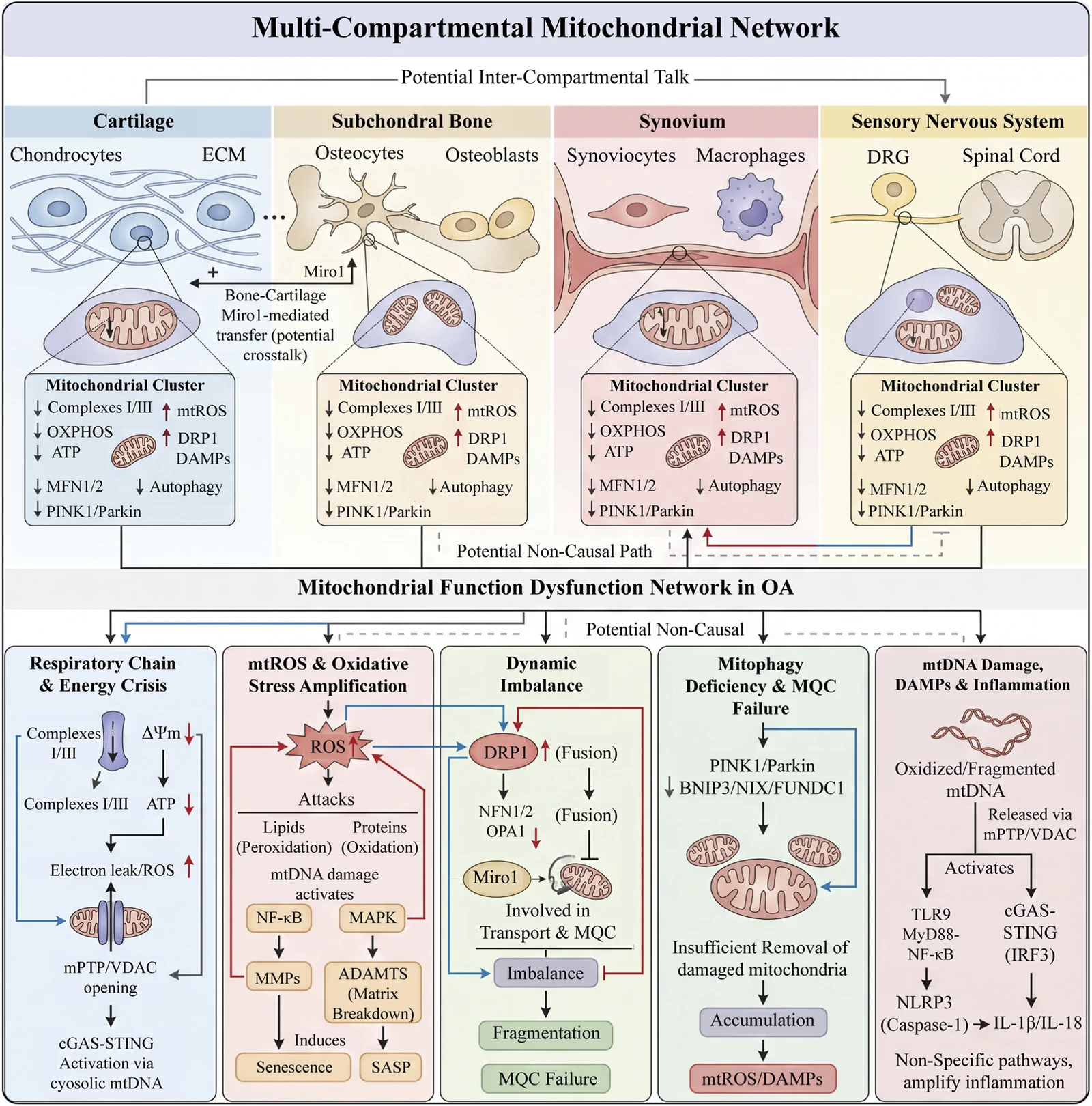
Multi-tissue compartment mitochondrial dysfunction network and potential cross-tissue crosstalk in osteoarthritis. This figure illustrates the mitochondrial abnormalities commonly present in cartilage, subchondral bone, synovium, and the sensory nervous system, and the interconnections among these tissues. OA-related mitochondrial damage is mainly manifested as decreased respiratory chain complex function, reduced OXPHOS and ATP production, elevated mtROS, mitochondrial dynamics imbalance, and impaired PINK1/Parkin-related mitophagy and disrupted mitochondrial quality control. This can further induce lipid and protein oxidation, mtDNA damage and release, and activate inflammatory pathways (e.g., NF-κB, MAPK, TLR9, NLRP3, and cGAS-STING), thereby promoting MMPs/ADAMTS-mediated matrix degradation, cellular senescence, and SASP formation. Cartilage–bone Miro1-mediated mitochondrial transfer and potential signaling between different tissue compartments may further amplify local inflammation and tissue degeneration, but some cross-compartment links currently remain speculative or non-causal.

## 4 Mitochondrial metabolic abnormalities and chondrocyte senescence

### 4.1 Chondrocyte senescence: a mitochondrial-related pathological node in OA progression

Chondrocytes are the only resident cells in articular cartilage, and their homeostasis is essential for the synthesis, turnover, and mechanical function of the ECM. During OA progression, chondrocytes gradually lose their matrix-synthesizing capacity and exhibit cell cycle arrest, upregulation of p16^INK4a^ and p21, enhanced senescence-associated β-galactosidase (SA-β-Gal) activity, and induction of the SASP (Jacob et al., 2022; Chen YH. et al., 2023). In OA research, “cell senescence” is often judged based on senescence-associated markers such as p16^INK4a^, p21, SA-β-Gal, and SASP, but these indicators are not absolutely specific. Therefore, in the absence of multi-marker combinations, persistent cell cycle arrest, and functional validation, it is more appropriate to describe this as a “senescence-associated phenotype” or “senescence-related changes,” rather than defining irreversible cell senescence based on a single marker. Unlike simple cellular aging, the senescence-associated chondrocyte phenotype in OA is often accompanied by mitochondrial dysfunction, enhanced oxidative stress, and imbalanced mitochondrial quality control, suggesting that it has prominent metabolic stress-related features (Zhang et al., 2024). Chondrocytes with a senescence-associated phenotype show decreased anabolic molecules such as COL2A1 and ACAN on the one hand, and increased SASP-related factors such as MMP-13, ADAMTS5, IL-6, IL-8, and CCL2 on the other (Swahn et al., 2023; Wang N. et al., 2022). These changes can disrupt cartilage matrix homeostasis and, through paracrine mechanisms, affect neighboring chondrocytes, synoviocytes, and immune cells, amplifying local inflammation and inter-tissue signaling communication (Liao et al., 2024; Steinhoff et al., 2022). Therefore, the chondrocyte senescence-associated phenotype may constitute an important pathological nexus linking mitochondrial abnormalities, matrix degeneration, and inflammation, but its direct causal relationship with pain persistence remains to be validated.

### 4.2 Potential mechanisms by which mitochondrial dysfunction participates in chondrocyte senescence

Existing studies indicate that mitochondrial dysfunction is closely associated with chondrocyte senescence in OA and may participate in the formation of the senescent phenotype through multiple interrelated processes (Xia et al., 2023). Among these, mtROS accumulation is a candidate mechanistic link that has received considerable attention. In OA, decreased respiratory chain function and increased electron leakage can lead to excessive mtROS generation (Sun et al., 2024). Excessive mtROS not only damages DNA, proteins, and lipids, but may also participate in the formation and maintenance of the chondrocyte senescence phenotype by affecting cell cycle arrest pathways such as p53/p21 and p16^INK4a^/Rb (Basilicata et al., 2025; Qin et al., 2025). At the same time, mtROS has also been linked to increased expression of SASP-related factors such as IL-6 and MMP-13 through downstream inflammatory and stress signaling pathways such as NF-κB and MAPK (Chu et al., 2023). Therefore, mtROS-related signaling may participate in cell cycle arrest and is intimately associated with SASP maintenance and ECM degradation, but its independent causal contribution to chondrocyte senescence in OA remains to be definitively established.

NAD^+^/Sirtuins abnormalities represent another important link connecting metabolic status and the senescent phenotype. SIRT1 mainly participates in cell cycle regulation, antioxidant responses, and mitochondrial biogenesis through factors such as p53, FOXO, and PGC-1α (Cui et al., 2022; Sun C. et al., 2023; Wu X. et al., 2025); in contrast, SIRT3 is mainly localized in the mitochondrial matrix and can directly regulate redox homeostasis by deacetylating mitochondrial proteins such as SOD2, and its functional decline is associated with reduced ROS scavenging capacity, decreased membrane potential, and insufficient ATP production (Zhang Y. et al., 2023; Wang X. et al., 2024). Therefore, SIRT3 more closely represents a direct mitochondrial regulatory factor, whereas SIRT1/PGC-1α operates within a mitochondrial-related metabolic regulatory network. These changes suggest that the NAD^+^/Sirtuins axis may participate in the link between mitochondrial homeostasis and chondrocyte senescence, but the cell type-specific roles of different Sirtuin members in OA and their causal hierarchy still need to be further clarified through interventional studies.

mtDNA damage may also participate in the amplification of senescence-related inflammatory responses through inflammatory nucleic acid-sensing pathways. In OA chondrocytes, mtROS accumulation can cause mtDNA oxidative damage, copy number abnormalities, and fragment release (Huang et al., 2026); after entering the cytoplasm, mtDNA can be recognized by nucleic acid-sensing pathways such as cGAS-STING and is associated with increased expression of type I interferons and inflammatory factors (Zhou et al., 2021). cGAS-STING is not a mitochondria-specific target, but rather a downstream immune pathway responding to mitochondria-derived DAMPs. In addition, ATP deficiency and impaired mitochondrial quality control may also perturb autophagy, mitophagy, and metabolic adaptation through energy-sensing networks such as AMPK/mTOR (Marzetti et al., 2024; Xiang et al., 2025; Wan et al., 2023). Therefore, chondrocyte senescence in OA is better understood as a complex phenotype interrelated with multiple processes, including mtROS accumulation, NAD^+^/Sirtuins abnormalities, mtDNA inflammatory sensing, energy metabolism disorders, and mitochondrial quality control failure, rather than being directly driven by a single mitochondrial abnormality. Existing evidence suggests that these mitochondrial-related changes may jointly participate in the formation and maintenance of the senescent phenotype, but their temporal relationships, relative contributions, and causal hierarchy remain to be validated.

### 4.3 SASP-mediated matrix degradation and potential cross-tissue effects

SASP is one of the key mechanisms by which senescence-associated chondrocytes, as observed in experimental studies, affect the local OA microenvironment. Senescence-associated chondrocytes can release inflammatory factors, chemokines, and matrix-degrading enzymes such as IL-6, IL-8, CCL2, TNF-α, MMP-13, and ADAMTS5 (Coryell et al., 2021). Among these, MMP-13 and ADAMTS5 participate in the degradation of type II collagen and aggrecan, respectively, and are important effector factors driving cartilage matrix destruction (Najar et al., 2020; Miyaji et al., 2021). At the same time, SASP factors can maintain or reinforce the senescence-associated phenotype in an autocrine manner and can also induce senescence-associated changes in neighboring chondrocytes through paracrine mechanisms, suggesting that the senescence-associated phenotype has a potential local spreading effect (Chen H. et al., 2022).

Beyond chondrocytes, independent studies on synovial fibroblasts and macrophages suggest that inflammatory mediators such as IL-6, CCL2, IL-1β, and TNF-α can participate in synovial inflammation and immune cell activation (Wang B. et al., 2024; Zhang S. et al., 2026). Mediators such as IL-1β, TNF-α, and PGE2 released by these cells may, in turn, affect chondrocytes, enhancing oxidative stress, SASP-related responses, and matrix degradation (Yan Z. et al., 2020; Cai et al., 2023). It should be emphasized that these findings derive mainly from studies of synovium and immune cells and do not yet establish a definitive causal role for SASP from senescent chondrocytes. Therefore, it is more appropriate to understand this as a potential inflammatory feedback among different joint compartments, rather than an established unidirectional causal chain of “chondrocyte SASP-synovial inflammation.”

On the other hand, sensory nerve-related studies indicate that inflammatory mediators such as IL-1β, TNF-α, IL-6, and PGE2 can affect nociceptive sensory pathways such as TRPV1, TRPA1, and P2X and are associated with peripheral sensitization (Kang et al., 2025; Lin et al., 2020; Jiang et al., 2020). These findings mainly support a mechanistic link between inflammatory mediators and sensory nerve sensitization, but do not establish that SASP derived from senescence-associated chondrocytes directly drives sensory nerve abnormalities. Mitochondrial abnormalities in sensory nerves and other joint compartments may also occur relatively independently. Therefore, the link between SASP and pain-related neuroimmune processes is currently more appropriately regarded as a potential cross-tissue mechanistic hypothesis, rather than an established continuous causal pathway of “cartilage degeneration-SASP-pain sensitization.”

### 4.4 Mechanical overload as a mitochondrial stressor in chondrocyte senescence

Abnormal mechanical loading is an important trigger for the development and progression of OA and is also a key external stimulus inducing chondrocyte mitochondrial damage and senescence. Mechanical loading within the physiological range helps maintain cartilage matrix homeostasis, whereas sustained or excessive mechanical stress can induce mitochondrial dynamics imbalance, mtROS accumulation, and energy metabolism disorders through changes in the cytoskeleton, mechanosensitive ion channels, and mitochondrial membrane tension (Shao et al., 2024; Zu et al., 2025). Under mechanical overload conditions, OA chondrocytes often exhibit enhanced DRP1-mediated mitochondrial fission, mitochondrial fragmentation, decreased membrane potential, and reduced ATP production, accompanied by activation of senescence pathways such as p53/p21 and p16^INK4a^ (Wang et al., 2026b). PDZ domain-containing protein 1 (PDZK1) may participate in the regulation of the interplay between mechanical stress, mitochondrial homeostasis, and chondrocyte senescence. Studies have shown that mechanical overload can lead to decreased PDZK1 expression in OA chondrocytes, and PDZK1 deficiency aggravates mitochondrial dysfunction, manifesting as accumulation of damaged mitochondria, reduced mtDNA content, increased ROS production, and enhanced chondrocyte senescence (Shao et al., 2024). Combined with interventional evidence, such as that from PDZK1 deficiency, these results support the notion that mechanical stress can participate in metabolic senescence of chondrocytes by disrupting mitochondrial homeostasis, rather than merely manifesting as direct ECM damage.

Targeting mitochondrial oxidative stress can partially block mechanical overload-induced chondrocyte senescence. In PDZK1-deficient or mechanically stretched chondrocytes, the mitochondria-targeted antioxidant MitoQ can protect mitochondrial function, reduce mtROS generation, and alleviate chondrocyte senescence while mitigating cartilage degeneration. In addition, highly migratory network hydrogel microspheres with mechanical buffering function can delay chondrocyte senescence and OA progression by dispersing mechanical stress, improving mitochondrial function, and inhibiting senescence-related pathways (Xu F. et al., 2025). These interventional studies further support a mechanistic link between mitochondrial abnormalities and the mechanical-load-induced chondrocyte senescence phenotype. Overall, mechanical stress, mitochondrial damage, senescence-related changes, and matrix degradation may form a mutually reinforcing pathological cycle, but their temporal relationships and relative contributions at different OA stages still need further clarification (Figure 3).

**FIGURE 3:**
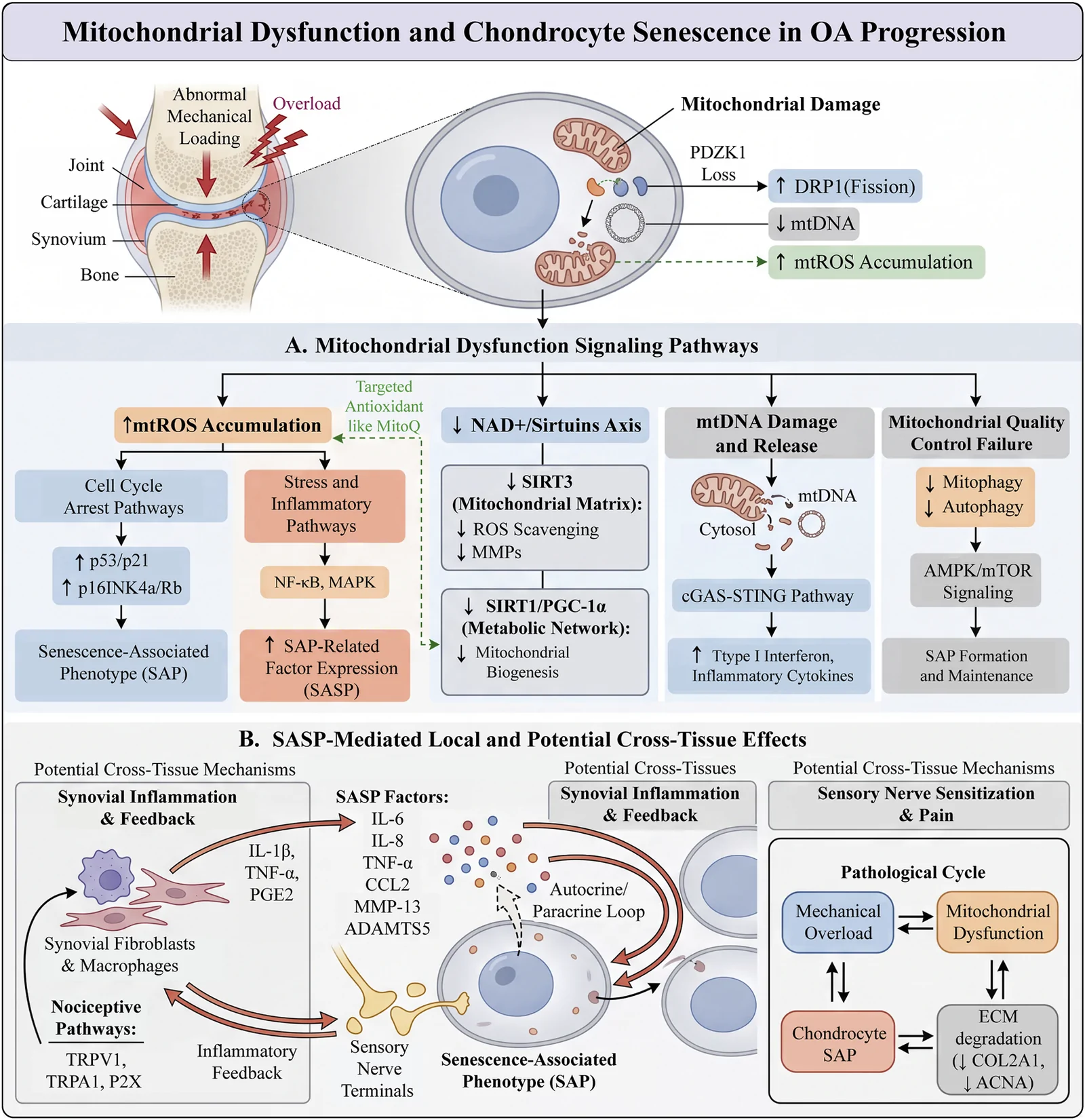
Mitochondrial dysfunction and chondrocyte senescence promote osteoarthritis progression. Abnormal mechanical loading can induce chondrocyte mitochondrial damage, manifested as PDZK1 deficiency-related enhanced DRP1-mediated mitochondrial fission, mtROS accumulation, and mtDNA abnormalities. **(A)** Mitochondrial dysfunction signaling pathways: elevated mtROS, impaired NAD^+^/Sirtuins axis, mtDNA damage and release, and mitochondrial quality control failure jointly activate pathways such as p53/p21, p16INK4a/Rb, NF-κB/MAPK, cGAS–STING, and AMPK/mTOR, promoting the chondrocyte SAP and the formation and maintenance of SASP. **(B)** SASP-mediated local and potential cross-tissue effects: senescent chondrocytes secrete SASP factors such as IL-6, IL-8, TNF-α, CCL2, MMP-13, and ADAMTS5, which through autocrine and paracrine effects amplify synovial inflammation, matrix degradation, and sensory nerve sensitization, and form a pathological cycle of “mechanical overload-mitochondrial dysfunction-chondrocyte senescence-ECM destruction,” driving sustained OA progression.

## 5 Mitochondrial metabolic abnormalities and regulated cell death

Mitochondrial dysfunction is closely associated with multiple forms of RCD and may participate in their occurrence and regulation, but these death programs are not all direct mitochondrial targets. Among them, MOMP, mPTP, and VDAC are directly related to mitochondrial structure and membrane function; in contrast, ferroptosis, pyroptosis, and PANoptosis-like signaling are more appropriately regarded as mitochondrial-related cell death pathways, whereas GPX4/System Xc^−^, NLRP3, and RIPK1/RIPK3/MLKL belong to the regulatory factors of the corresponding death programs (Niu et al., 2026). Existing evidence suggests that mitochondrial abnormalities are closely related to OA-associated cell death, oxidative stress, and inflammatory responses, and may participate in the amplification of these pathological processes; however, the causal direction and relative contribution between different RCD forms and mitochondrial abnormalities may depend markedly on cell type and disease stage. Therefore, these associations alone are insufficient to prove that mitochondrial dysfunction constitutes a continuous mechanistic bridge from cartilage degeneration to pain sensitization.

### 5.1 Mitochondrial pathway-mediated apoptosis

Excessive chondrocyte death is an important pathological basis for progressive OA cartilage loss and disruption of matrix homeostasis, among which mitochondrial pathway-mediated apoptosis is one of the more thoroughly studied forms of RCD (He B. et al., 2020). Oxidative stress, inflammatory factors, and abnormal mechanical stress in the OA microenvironment can induce mitochondrial outer membrane permeabilization (MOMP), promoting the release of pro-apoptotic factors such as cytochrome c into the cytoplasm (Dadsena et al., 2021; Ansari et al., 2021). Cytochrome c subsequently forms an apoptosome with apoptotic protease activating factor-1 (Apaf-1) and activates caspase-9, further initiating the caspase-3/7 cascade, ultimately leading to DNA fragmentation and apoptosis (Wang H. et al., 2020; Li Z. et al., 2020). In OA cartilage tissue, caspase-3 activity is elevated and correlates with the degree of cartilage degeneration, supporting the involvement of mitochondrial apoptosis in chondrocyte loss and disease progression (Wang et al., 2021).

The Bcl-2 family is the core regulatory system of MOMP. Oligomerization of the pro-apoptotic proteins Bax and Bak can increase mitochondrial outer membrane permeability, whereas Bcl-2 and Bcl-xL maintain mitochondrial integrity by inhibiting this process (Rühl et al., 2025). Under OA conditions, an increased Bax/Bcl-2 ratio, decreased Bcl-2 expression, and enhanced Bax mitochondrial translocation can render cells more susceptible to the intrinsic apoptotic program (Zhang et al., 2021; Darweesh et al., 2021). In addition to Bax/Bak-mediated MOMP, persistent opening of the mPTP and functional abnormalities of VDAC may also amplify this process (He et al., 2022). Persistent mPTP opening can lead to loss of membrane potential, mitochondrial swelling, and ATP depletion, and together with disruption of outer membrane integrity, promotes the release of pro-apoptotic signals (Korotkov, 2023); VDAC can influence outer membrane permeability and cell fate by regulating metabolite and Ca^2+^ exchange and through interactions with Bcl-2 family proteins (Wang and Liu, 2020). Therefore, mPTP/VDAC-mediated membrane permeability abnormalities and Bcl-2 family regulation do not act independently, but may jointly convert ROS, Ca^2+^ imbalance, and energy stress into mitochondrial apoptotic signals.

In addition, mitochondria-associated proteins such as BNIP3 may also participate in this regulatory network, and their abnormal expression may further disrupt mitochondrial homeostasis and promote chondrocyte death (Wang WF. et al., 2020). Therefore, mitochondrial apoptosis can be regarded as an important cell death pathway after the convergence of mechanical stress, oxidative stress, and inflammatory stimuli in OA; persistent or excessive activation will deplete functional chondrocytes, weaken matrix repair capacity, and ultimately accelerate cartilage degeneration.

### 5.2 Ferroptosis

Ferroptosis is an iron-dependent form of RCD driven by lipid peroxidation, and existing research suggests that ferroptosis-related changes and increased ferroptotic susceptibility may participate in OA chondrocyte damage and cartilage degeneration (Xu Y. et al., 2023). In OA chondrocytes, iron metabolism disorders are considered to be closely related to increased ferroptotic susceptibility (Yao et al., 2021). It should be noted that current support for ferroptosis in OA research mainly comes from increased iron load, lipid peroxidation, abnormalities in the GPX4/SLC7A11 defense system, mitochondrial morphological changes, and some interventional studies, but not all studies simultaneously use multidimensional indicators and functional validation. Therefore, when primarily based on molecular markers or correlative changes, it is more appropriate to describe them as “ferroptosis-related changes” or “increased ferroptotic susceptibility,” rather than concluding that a complete ferroptotic process has occurred based on a single indicator alone. Existing studies have shown that iron content is elevated in OA chondrocytes, accompanied by upregulation of transferrin receptor 1 (TFR1) and downregulation of ferritin heavy chain (FTH), leading to expansion of the intracellular labile iron pool (LIP) (Wang et al., 2026c; Guan et al., 2023). Excess free iron can promote ROS generation through the Fenton reaction and attack polyunsaturated fatty acids in cell membranes, triggering a lipid peroxidation chain reaction and leading to the accumulation of oxidative products such as 4-hydroxynonenal (4-HNE) and malondialdehyde (MDA) (Liu et al., 2024b; Chen Z. et al., 2025).

Glutathione peroxidase 4 (GPX4) and the cystine/glutamate antiporter system (System Xc^−^) are core components of the ferroptosis defense system. GPX4 can reduce phospholipid hydroperoxides to non-toxic phospholipid alcohols, thereby limiting the propagation of lipid peroxidation (Xie et al., 2023); while SLC7A11, a key subunit of System Xc^−^, provides the basis for GPX4 activity by maintaining cysteine uptake and glutathione (GSH) synthesis (Miao et al., 2022). Under OA conditions, decreased expression of SLC7A11 and GPX4 can weaken the cell’s ability to resist lipid peroxidation and is associated with lipid ROS accumulation and increased ferroptotic susceptibility (Wang et al., 2026c). Therefore, iron overload, impairment of the SLC7A11/GSH/GPX4 defense axis, and enhanced lipid peroxidation can be regarded as important features of ferroptosis-related phenotypes and increased susceptibility in OA chondrocytes (Wu B. et al., 2025; Xiao et al., 2024). In experimental models with pharmacological or genetic intervention, these results can further support the involvement of ferroptosis in chondrocyte damage, but its causal contribution and disease stage specificity in human OA still require further validation.

Mitochondria may play an important regulatory role in ferroptosis. On one hand, the mitochondrial electron transport chain is an important source of ROS, and respiratory chain dysfunction may further enhance iron-dependent lipid peroxidation (Javadov, 2022); on the other hand, mitochondria participate in iron-sulfur cluster and heme biosynthesis, and dysregulation of mitochondrial iron homeostasis can affect redox balance and energy metabolism. Mitochondrial iron overload can damage iron-sulfur cluster proteins and respiratory chain complexes, increasing electron leakage and ROS generation (Read et al., 2021; Zhao et al., 2024). In addition, abnormalities in iron transport-related molecules such as DMT1 and enhanced ferritinophagy may promote free iron release and increase mitochondrial iron load, thereby aggravating lipid peroxidation (Zhao G. et al., 2026). Morphological changes such as reduced mitochondrial cristae, decreased membrane potential, and mitochondrial shrinkage appear simultaneously with ferroptosis-related phenotypes in some experimental models, but these mitochondrial morphological and functional changes themselves are not specific criteria for confirming ferroptosis (Lu et al., 2026). Overall, these findings support a close connection between ferroptosis-related changes in OA and mitochondrial iron metabolism, respiratory chain function, and oxidative stress networks, but they are insufficient to define broad mitochondrial dysfunction as a single causal driver of OA ferroptosis. Therefore, ferroptosis should still be regarded as a regulated cell death program that interacts with mitochondrial function, rather than a mitochondria-specific cell death mechanism.

### 5.3 Pyroptosis and inflammasomes

Pyroptosis is an inflammatory RCD characterized by inflammasome activation, Gasdermin D (GSDMD)-mediated membrane pore formation, and release of pro-inflammatory cytokines (Liang et al., 2020). In OA, the NLRP3 inflammasome can be activated in chondrocytes, synoviocytes, and synovial macrophages, and is closely related to mitochondrial dysfunction (Chen B. et al., 2022; Broz, 2025). Mechanical overload, oxidative stress, and inflammatory stimuli can lead to a decrease in mitochondrial membrane potential, the opening of the mitochondrial permeability transition pore, and the release of mitochondrial reactive oxygen species (mtROS) and mtDNA (Pan and Zhu, 2026). These mitochondria-derived danger signals can serve as DAMPs and may participate in the priming and activation of the NLRP3 inflammasome.

Upon activation, the NLRP3 inflammasome can recruit ASC and pro-caspase-1, forming an inflammasome complex and activating caspase-1 (Zhang Z. et al., 2023). Activated caspase-1, on one hand, cleaves pro-IL-1β and pro-IL-18, maturing them into IL-1β and IL-18; on the other hand, it cleaves GSDMD, releasing its N-terminal domain (GSDMD-NT) (Huang et al., 2025). GSDMD-NT oligomerizes on the cell membrane to form pores, leading to release of cellular contents and inflammatory cell death (Linder and Hornung, 2020). In OA patient cartilage tissue, expression of pyroptosis-related proteins such as NLRP3, caspase-1 p20, and GSDMD-NT is elevated and correlates positively with the degree of cartilage degeneration, suggesting that pyroptosis may participate in chondrocyte damage and local inflammatory amplification (Xu L. et al., 2026). Of note, increased expression or activation of NLRP3, caspase-1, or GSDMD-related molecules primarily indicates enhanced inflammasome and pyroptosis-related signaling, and does not always equate to proven execution of a complete pyroptotic process. Therefore, in the absence of multidimensional evidence including membrane pore formation, cell lysis, and functional intervention, it is more appropriate to adopt qualifiers such as “pyroptosis-related signaling” or “pyroptosis-associated changes.”

Mitochondria-derived stress signals may participate in the activation of the NLRP3 inflammasome and pyroptosis-related signaling and the amplification of inflammatory feedback. Among these, increased mtROS, mtDNA release, and insufficient clearance of damaged mitochondria have all been reported to be associated with NLRP3 inflammasome activation. MtROS can be associated with NLRP3 conformational changes and ASC oligomerization, mtDNA release can enhance pattern recognition receptor-mediated sterile inflammatory responses, and insufficient mitophagy prevents timely clearance of damaged mitochondria, which may further maintain or enhance NLRP3 inflammasome activation (Li X. et al., 2024). In addition to chondrocytes, mitochondrial dysfunction in synovial macrophages is also associated with inflammasome activation and maintenance of the pro-inflammatory phenotype. For example, interventional studies have suggested that PIM-1-related signaling can enhance ASC oligomerization by promoting mtROS production and chloride efflux, thereby facilitating NLRP3 inflammasome assembly (Zhang Z. et al., 2023). Therefore, existing evidence supports a relatively close mechanistic link between mitochondria-derived stress signals, NLRP3 inflammasome activation, and pyroptosis-related changes in OA, suggesting they may participate in regulating and amplifying local inflammatory responses. However, the cell models, molecular markers, and degree of functional validation used across studies are not consistent; thus, not all inflammasome-related changes can be equated with complete pyroptosis. At the same time, as a broad concept, the independent causal contribution of “mitochondrial dysfunction” in the above processes still needs to be further clarified through cell type-specific intervention studies. Related changes in different cellular compartments may jointly participate in the OA inflammatory microenvironment, but a continuous causal pathway extending from chondrocyte death to pain sensitization cannot be established on this basis alone.

### 5.4 PANoptosis-like RCD cross-talk

In addition to single forms of RCD, cross-activation of multiple cell death pathways may exist in OA chondrocytes. PANoptosis is an inflammatory cell death concept proposed in recent years, emphasizing that apoptosis, pyroptosis, and necroptosis-related molecules can intertwine in the same pathological environment and may be coordinately regulated by PANoptosome-like complexes (Li H. et al., 2026; Li P. et al., 2025). In OA research, caspase-3 activation, enhanced NLRP3/caspase-1 signaling, and elevated RIPK3/MLKL phosphorylation can be simultaneously detected in chondrocytes (Malireddi et al., 2020). These findings suggest that OA chondrocyte death is not necessarily dominated by a single pathway, but may manifest as cross-talk among multiple RCD pathways. However, it remains premature to classify cell death in OA as classical PANoptosis (Zeng et al., 2021). Existing evidence more robustly supports the more cautious term “PANoptosis-like RCD cross-talk,” meaning that mechanical stress, inflammatory factors, mtROS accumulation, and mtDNA/DAMPs release in the OA microenvironment may accompany or participate in the co-activation of apoptosis, pyroptosis, and necroptosis-related signals. However, whether complete PANoptosome complex formation exists, the temporal relationship among different death pathways, and cell type-specific regulatory mechanisms remain to be fully elucidated (Smallwood et al., 2024). Therefore, PANoptosis-like RCD cross-talk is more suitable as a hypothetical integrative framework or working hypothesis for explaining the complexity of OA cell death, rather than a fully proven independent cell death mechanism.

Mitochondrial dysfunction is closely related to the cross-activation of multiple RCD pathways and may participate in their regulation through mtROS, membrane permeability changes, mtDNA/DAMPs release, and energy metabolism abnormalities. There is a mechanistic link between mtROS and caspase-3 activation and NLRP3 inflammasome assembly; decreased mitochondrial membrane potential and MOMP can induce cytochrome c release and initiate intrinsic apoptosis (Merlini et al., 2022; Pérez et al., 2024); mtDNA and other DAMPs release can amplify sterile inflammation; energy metabolism disorders and oxidative stress may also affect RIPK1/RIPK3/MLKL-related necroptotic signaling (Bryant et al., 2022; Ding et al., 2025). These mechanisms do not imply that mitochondrial dysfunction occupies a unified and fixed “upstream” position in all RCD forms, but more likely reflect context-dependent bidirectional or cross-regulation between mitochondrial stress and different cell death programs. These findings support the involvement of mitochondrial abnormalities in OA-related cell death heterogeneity and inflammatory amplification. However, the evidence in this section primarily supports an association and mechanistic link between “mitochondrial dysfunction-cell death/inflammation,” and does not permit the inference that mitochondrial abnormalities are an inevitable mechanistic bridge connecting cartilage degeneration and pain sensitization. In particular, there is still insufficient evidence to prove that a single unified mitochondrial abnormality can sequentially drive multiple RCDs and further lead to the OA pain phenotype. For evidence primarily obtained from molecular marker co-expression or experimental models, it should be interpreted as RCD-related signaling or putative cross-talk, rather than an established unified cell death program. Future research still needs to clarify the relative contributions of different RCD forms at different OA stages and in different cell types, their temporal relationships, and the causal direction of their relationship with mitochondrial abnormalities, thereby providing more precise evidence for mechanism-oriented interventions (Figure 4).

**FIGURE 4:**
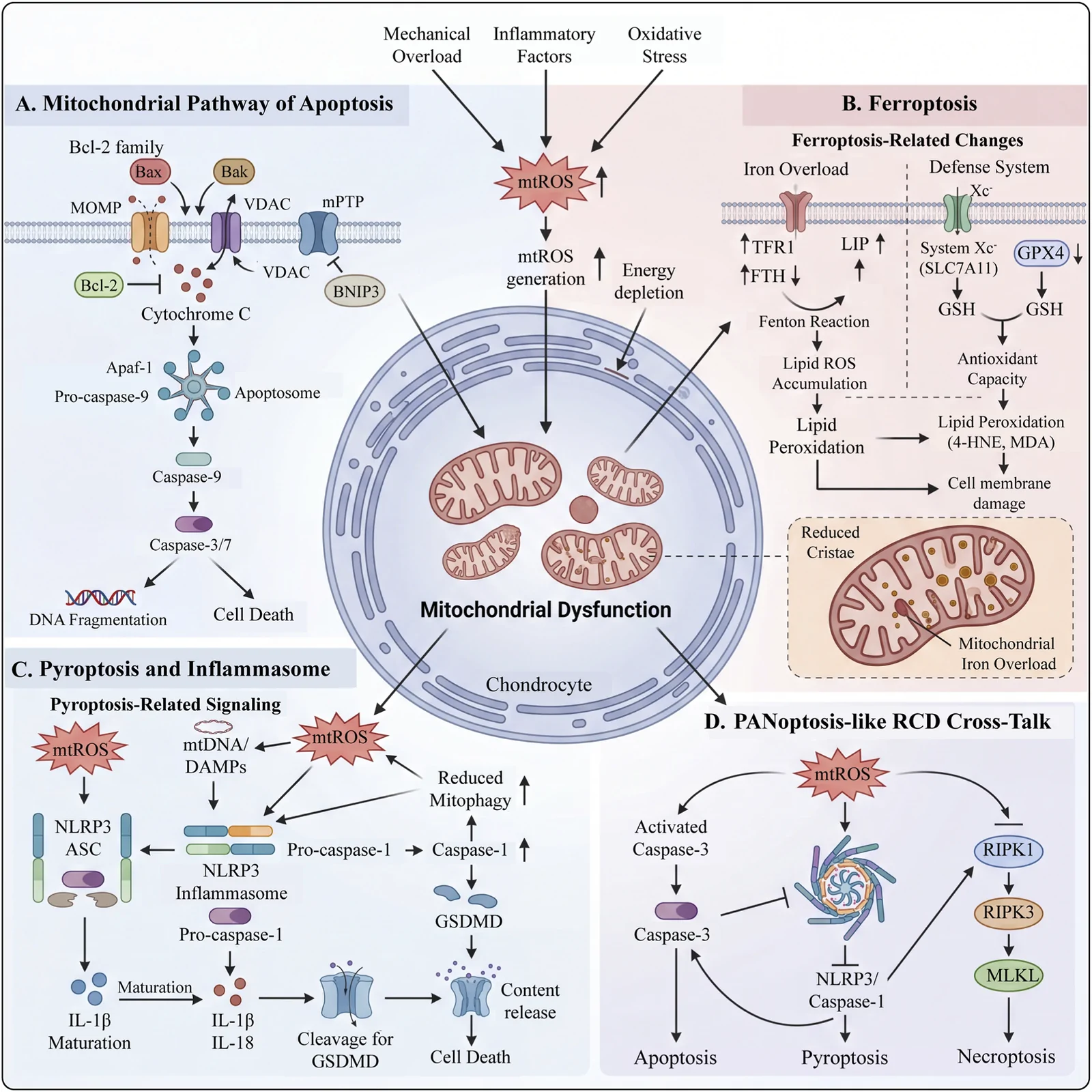
Mitochondrial dysfunction mediates multiple regulated cell death modes and their cross-talk in chondrocytes. Mechanical overload, inflammatory factors, and oxidative stress can induce chondrocyte mitochondrial damage, mtROS accumulation, and energy depletion, and further activate multiple regulated cell death pathways. **(A)** Mitochondrial apoptosis: Bcl-2 family proteins regulate MOMP, VDAC, and mPTP opening, promoting cytochrome c release and the Apaf-1/caspase-9/caspase-3/7 cascade, ultimately leading to DNA fragmentation and cell death. **(B)** Ferroptosis: Increased iron load, enhanced Fenton reaction, and impairment of the System Xc^−^/GSH/GPX4 antioxidant defense jointly promote lipid peroxidation and membrane damage. **(C)** Pyroptosis and inflammasomes: mtROS, mtDNA, and DAMPs can activate the NLRP3 inflammasome and caspase-1, inducing GSDMD cleavage and maturation and release of IL-1β/IL-18. **(D)** PANoptosis-like RCD cross-talk: Apoptosis, pyroptosis, and necroptosis-related molecules, including caspase-3, NLRP3/caspase-1, and RIPK1/RIPK3/MLKL, may undergo mutual regulation under persistent mitochondrial stress, thereby collectively amplifying chondrocyte damage.

## 6 Mitochondrial dysfunction and OA pain sensitization

### 6.1 OA pain is not entirely equivalent to structural damage

OA pain and joint structural damage do not always exhibit a linear or consistent correlation. Clinical studies indicate that cartilage degeneration, joint space narrowing, and osteophyte formation observed on X-ray or MRI often correlate only weak to moderate with patients’ subjective pain scores (Pan et al., 2020; Chung et al., 2023). Some patients present with significant structural damage but mild pain symptoms, whereas others with only mild to moderate imaging changes may experience persistent, intractable pain (Henningsen et al., 2018). This “structural damage-pain mismatch” phenomenon suggests that OA pain cannot be explained solely by cartilage wear or abnormal mechanical loading; it also involves multi-level mechanisms such as synovial inflammation, subchondral bone remodeling, bone marrow lesions, abnormal neurovascularization, and peripheral and central pain sensitization (Ohashi et al., 2023; Vincent and Miller, 2024).

Pain sensitization is an important mechanism explaining this structural-symptom decoupling. Peripheral sensitization primarily manifests as enhanced responsiveness of local joint nociceptors to inflammatory and metabolic stimuli, whereas central sensitization manifests as amplified processing of persistent nociceptive input by the spinal cord and higher centers (Steen Pettersen et al., 2020; Aoyagi et al., 2026). Existing evidence suggests that mitochondrial dysfunction may exist separately in structural joint lesions and pain-related neural compartments, and it should not be presumed that the former necessarily drives the latter (Silva Santos Ribeiro et al., 2022). Mitochondrial abnormalities in cartilage, synovium, subchondral bone, and the sensory nervous system may interact through inflammatory and metabolic signals such as SASP, mtROS, mtDNA, and other DAMPs, and participate in local nociceptive input and neural sensitization-related processes (Zheng L. et al., 2021; Antonucci et al., 2021). It should be emphasized that even if elevated mtROS, decreased ATP, abnormal membrane potential, or impaired mitochondrial quality control are observed in both chondrocytes and sensory neurons, these similar phenotypes should not be regarded as continuous manifestations of the same mitochondrial pathological process in different compartments, nor should it be inferred that the same mitochondrial abnormality simultaneously drives structural OA progression and pain sensitization (Mao et al., 2026). Existing evidence better supports that these changes may represent relatively independent but partially shared stress responses under different cellular contexts, unless cell-specific interventions or longitudinal studies demonstrate a common causal origin. However, there is currently insufficient evidence to prove that chondrocyte mitochondrial abnormalities are the necessary mechanistic bridge linking cartilage degeneration and pain sensitization; mitochondrial abnormalities in sensory nerves and the central nervous system may also exist as relatively independent pathological processes. Therefore, this article regards pain sensitization as a core dimension that is interrelated with tissue damage but has a relatively independent mechanistic basis, and separately examines mitochondrial abnormalities in joint structure-related compartments and pain-related neural compartments and their potential interactions, without presupposing that both are driven by the same mitochondrial abnormality.

### 6.2 Potential mechanisms by which mitochondrial damage participates in peripheral pain sensitization

Peripheral pain sensitization occurs in local joint tissues and their sensory nerve endings. This process plays a critical role in the formation of OA pain. Evidence from cartilage- and synovium-related studies suggests that under mitochondrial stress, chondrocytes and synoviocytes can release IL-1β, IL-6, TNF-α, PGE2, CCL2, and mitochondria-derived DAMPs. In addition to participating in cartilage matrix degradation and synovial inflammation, these mediators may alter the inflammatory and metabolic microenvironment surrounding nociceptors (Peng et al., 2021). Among them, mitochondrial danger signals such as mtDNA, ATP, and N-formyl peptides can enhance sensory nerve ending excitability through pathways such as TLR9, P2X7 receptors, and formyl peptide receptors, suggesting that local joint mitochondrial stress may participate in the formation of a pain-related microenvironment, but such evidence is still insufficient to prove that cartilage mitochondrial damage itself directly drives sensory nerve sensitization (Rebetz et al., 2025). It should be emphasized that these findings mainly reflect potential links between signals derived from local joint tissues and nociceptive sensory pathways. However, this evidence cannot prove that chondrocyte mitochondrial damage directly leads to sensory neuron mitochondrial dysfunction or peripheral sensitization (Li X. et al., 2025). At this level, mtROS, mtDNA, abnormal ATP production, and mPTP/VDAC-mediated membrane functional changes belong to mitochondrial intrinsic or mitochondria-derived factors, whereas TRPV1, TRPA1, P2X7, TLR9, and other nociceptive and immune signals mainly belong to their downstream effector pathways (Zheng B. et al., 2021; Chappell et al., 2020; Chappell et al., 2021).

Unlike the above evidence from local joint tissues, studies on DRG sensory neurons mainly support a link between mitochondrial abnormalities in sensory neurons themselves and peripheral sensitization. In OA animal models, DRG neurons can exhibit decreased mitochondrial membrane potential, insufficient ATP supply, and elevated mtROS, accompanied by increased sensitivity of ion channels such as TRPV1 and TRPA1 and mechanical or thermal hyperalgesia (Wang et al., 2025). ATP depletion may impair Na^+^/K^+^-ATPase-mediated membrane potential maintenance, leading to increased abnormal firing. Additionally, presynaptic mitochondrial damage may disrupt vesicle cycling and neurotransmitter release (Chen et al., 2026). In this process, Ca^2+^ overload and oxidative stress-related mPTP opening may further aggravate loss of membrane potential and ATP depletion, and abnormal VDAC-mediated metabolite and Ca^2+^ exchange may also affect neuronal excitability (Mohsin et al., 2020). These results support an association between DRG neuronal mitochondrial dysfunction and pain sensitization. However, whether these neuronal mitochondrial abnormalities are secondary to mitochondrial damage in cartilage, synovium, or other joint tissues remains uncertain.

At present, OA-specific causal evidence that mPTP/VDAC and some mitochondrial stress mechanisms directly participate in OA pain sensitization remains relatively limited. Existing studies suggest that abnormalities in the circHIPK2/miR-206/SIRT3 axis are associated with mitochondrial metabolic imbalance and mechanical hyperalgesia in the context of OA, providing some support for a link between sensory neuron metabolic abnormalities and pain (Shen et al., 2026). In addition, non-OA models such as chemotherapy-related neuropathic pain, diabetic peripheral neuropathy, and fibromyalgia also suggest that mtROS accumulation, mtDNA release, and TRP channel sensitization may participate in peripheral pain amplification (Ho et al., 2025). These non-OA models only provide mechanistic references and cannot replace direct evidence from OA joint tissues or DRG sensory neurons, nor can they be used to establish a continuous causal chain of “joint tissue mitochondrial damage-DRG mitochondrial abnormalities-pain sensitization.”

### 6.3 Subchondral bone mitochondrial abnormalities and bone pain-like sensitization

Subchondral bone, which is rich in blood vessels and nerve endings, serves as an important source of OA pain. Abnormal subchondral bone remodeling can occur in the early stage of OA, including enhanced osteoclast activity, increased bone resorption, microfracture formation, and bone marrow lesions (Rösch et al., 2023; Zhao et al., 2022). Mitochondrial abnormalities in osteoblasts and osteoclasts are closely related to imbalanced bone remodeling. Specifically, elevated mtROS levels may promote osteoclast differentiation and activation, contributing to bone resorption and microstructural changes (He et al., 2025).

Osteoclast overactivation can also form a local acidic microenvironment, activating sensory nerve endings through acid-sensing ion channels (ASICs) and P2X receptors (Zhu et al., 2026). At the same time, abnormal bone remodeling is often accompanied by increased neurovascularization and extension of nerve fibers toward the osteochondral junction. These changes are closely related to bone-derived pain (Okamoto et al., 2017; Boyce and Xing, 2007). Among them, ASICs and P2X are categorized as pain-related effector pathways rather than direct mitochondrial targets. Therefore, mitochondrial abnormalities in subchondral bone may contribute to bone pain-like sensitization by influencing bone remodeling, the local metabolic environment, and neurovascular changes. However, this process may represent a pathological mechanism intrinsic to the subchondral bone compartment and does not require mediation by chondrocyte mitochondrial abnormalities. Existing evidence is also insufficient to define it as an obligatory causal link in the transformation from cartilage structural degeneration to pain.

### 6.4 Mitochondrial dysfunction and central sensitization

Persistent peripheral nociceptive input can induce plastic changes in the spinal dorsal horn and higher centers, namely, central sensitization. Chronic pain models of OA suggest that spinal dorsal horn neurons can exhibit decreased mitochondrial membrane potential, reduced ATP production, and elevated ROS, accompanied by enhanced neuronal excitability (Chen and Tang, 2024). Abnormal mitochondrial energy metabolism may affect glutamate reuptake and synaptic vesicle cycling, enhancing glutamatergic signaling; subsequent overactivation of NMDA receptors and Ca^2+^ influx further aggravate mitochondrial Ca^2+^ load, forming a positive feedback loop of “neuronal excitation-Ca^2+^ overload-mitochondrial damage” (Mira and Cerpa, 2021; Lin et al., 2025). In this context, persistent mPTP opening and abnormal VDAC-related Ca^2+^ flux may also amplify neuronal mitochondrial stress, but their direct role in OA central sensitization still needs verification. These findings mainly support a mechanistic association between mitochondrial abnormalities in the central nervous system itself and pain maintenance, and cannot be used to inversely infer that they originate from mitochondrial damage in cartilage or other joint tissues.

Microglia also participate in this process. Persistent peripheral input and DAMPs can induce spinal microglial activation, accompanied by decreased mitochondrial membrane potential, reduced ATP, and increased mtROS (Karavis et al., 2023). Damaged mitochondria may promote maintenance of a proinflammatory phenotype and release of mediators such as TNF-α and IL-1β, and further affect dorsal horn neuronal excitability; mitochondrial DAMPs such as mtDNA can also amplify neuroinflammation through pattern recognition receptors (Liu et al., 2022; Liu et al., 2024c). These inflammatory and immune pathways may be considered downstream effects of mitochondrial stress rather than direct mitochondrial mechanisms. Therefore, mitochondrial dysfunction may contribute to OA central sensitization and chronic pain maintenance via abnormal neuronal energy metabolism, disturbed Ca^2+^ homeostasis, and glia-mediated inflammatory responses (Zhang et al., 2026b; Zhao Q. et al., 2026). However, current relevant evidence is still mainly based on animal models and mechanistic studies, and its temporal relationship and independent causal contribution in human OA central sensitization still need further clarification.

Overall, mitochondrial abnormalities may exist simultaneously in local joint tissues, sensory neurons, and the spinal neuro-glial system. Among them, mtROS, mtDNA, abnormal ATP production, and mPTP/VDAC-related membrane functional changes represent mitochondrial intrinsic or mitochondria-derived abnormalities, whereas TRP/P2X, TLR9, NMDA, and inflammatory factors mainly constitute their downstream neural or immune effector networks. Existing evidence better supports that mitochondrial dysfunction may separately participate in structural OA pathology and different levels of pain sensitization, rather than having already proven that chondrocyte mitochondrial abnormalities are a single mechanistic bridge between the two. Mitochondrial abnormalities in different compartments may occur in parallel and may also undergo limited cross-compartment interactions through metabolic, inflammatory, and neuroimmune signals, but their temporal sequence and causal direction remain unclear. Therefore, this multi-compartment framework is more suitable as a potential mechanistic model to explain the inconsistency between OA structural damage and pain severity, rather than as an established continuous causal pathway. Accordingly, interventions that alleviate pain sensitization cannot be directly regarded as having structural disease-modifying effects on this basis; similarly, structural protection cannot automatically be inferred to have analgesic effects. This distinction is especially important to make when evaluating mitochondria-related therapeutic strategies (Figure 5).

**FIGURE 5:**
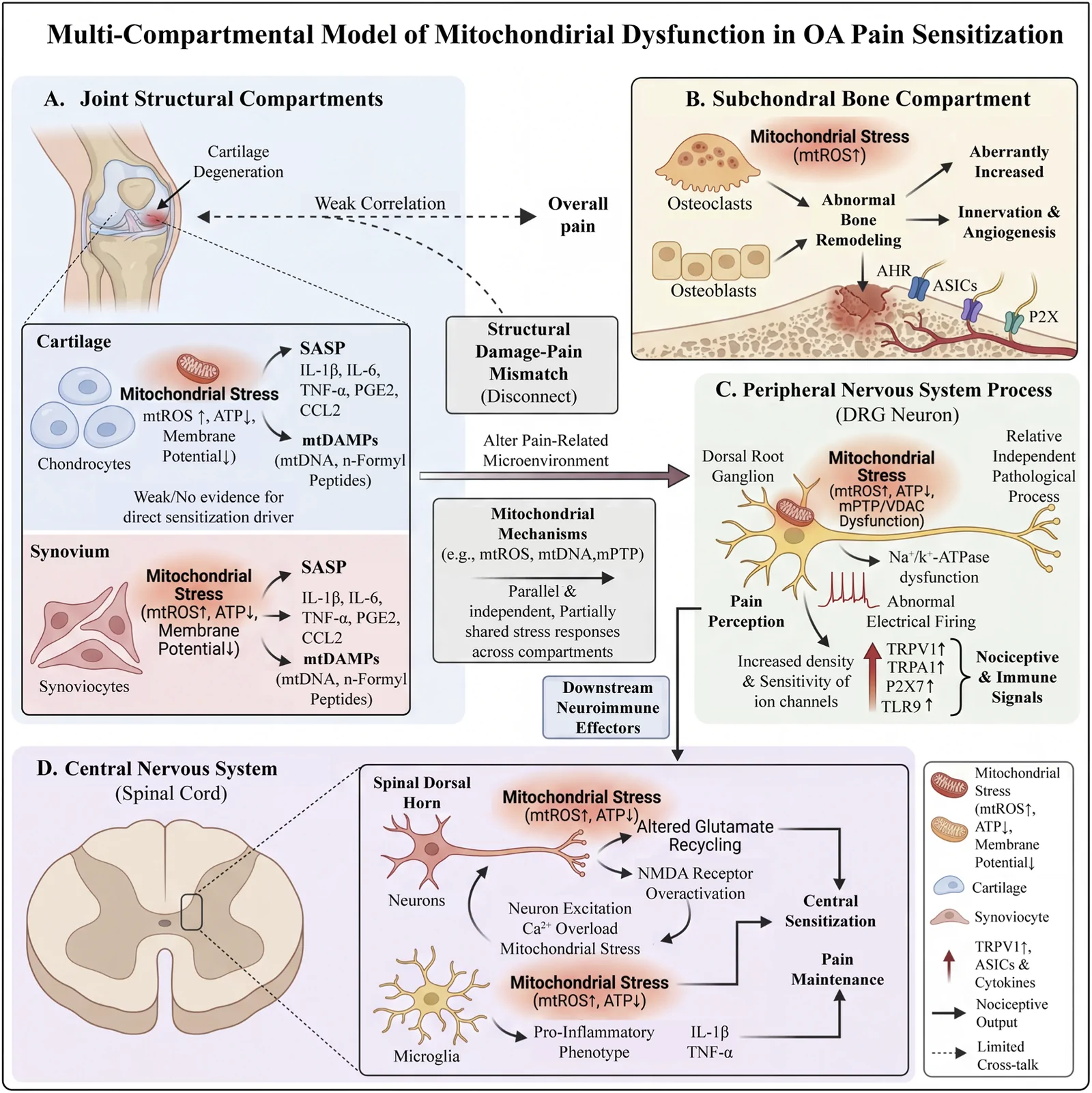
A multi-compartment mechanistic model of mitochondrial dysfunction participating in osteoarthritis pain sensitization. This figure outlines the potential links between mitochondrial stress in joint structures, subchondral bone, peripheral sensory nerves, and the central nervous system and their contribution to the formation and maintenance of OA pain. **(A)** Joint structure compartment: Mitochondrial stress in chondrocytes and synoviocytes manifests as elevated mtROS, decreased ATP, and reduced membrane potential, accompanied by release of SASP and mtDAMPs, which in turn alters the local pain-related microenvironment; however, the correlation between structural damage and pain severity is limited. **(B)** Subchondral bone compartment: Mitochondrial stress in osteoblasts and osteoclasts can promote abnormal bone remodeling, accompanied by increased angiogenesis and innervation, thereby enhancing local nociceptive signals. **(C)** Peripheral nervous system: Mitochondrial dysfunction in DRG neurons can cause impaired energy metabolism, abnormal Na^+^/K^+^-ATPase activity, and abnormal neuronal firing, which enhances nociceptive/immune signals such as TRPV1, TRPA1, P2X7, and TLR9, thereby promoting peripheral sensitization. **(D)** Central nervous system: Mitochondrial stress in spinal neurons and microglia can promote central sensitization and pain maintenance through abnormal glutamate homeostasis, NMDA receptor overactivation, Ca^2+^ overload, and proinflammatory responses. Different compartments may have parallel or partially shared mitochondrial stress mechanisms, but some cross-tissue links currently lack direct causal evidence.

## 7 Mitochondria-targeted therapeutic strategies: from preclinical evidence to translational application

As the role of mitochondrial dysfunction in OA structural degeneration and pain sensitization has garnered increasing attention, regulating mitochondrial homeostasis has become an important direction for mechanism-oriented intervention. However, improvement after therapeutic intervention itself does not prove that mitochondrial dysfunction is the primary causal driver of OA structural degeneration or pain. Therefore, this article distinguishes mitochondria-directed strategies that act directly on mitochondrial structure or function from mitochondria-related strategies that indirectly affect mitochondrial homeostasis through metabolic, inflammatory, aging, or cell death pathways. When evaluating translational evidence, a distinction should also be made among mechanistic proof, therapeutic efficacy, and biomarker response: target-specific intervention, blockade, or rescue experiments can support mechanistic causality; structural, pain, and functional outcomes are used to evaluate therapeutic efficacy; whereas changes in indicators such as mtROS, ATP, mitochondrial membrane potential, or mtDNA mainly reflect biological or pharmacodynamic responses and cannot, on their own, prove that a mechanism is established or that treatment is effective. At the same time, structural disease-modifying effects and analgesia/symptom improvement should be evaluated as two independent but complementary dimensions of efficacy. Structural protection cannot be directly equated with analgesic effect, and pain improvement cannot alone serve as evidence of disease-modifying effects. Even if the same intervention simultaneously improves structural outcomes, pain behavior, and mitochondria-related indicators, this cannot prove that these effects are mediated by the same mitochondrial mechanism. Unless specific intervention, mechanistic blockade, or rescue evidence is obtained for pain-related cells or neural pathways, improvement in mitochondrial indicators should not be directly interpreted as an analgesic mechanism. Therefore, the following sections will separately discuss the mechanisms of action, structure-related evidence, pain/symptom evidence, and translational maturity of different interventions, with particular attention to possible differences in mitochondrial phenotypes and therapeutic responses across different cells and tissue compartments (Figure 6).

**FIGURE 6:**
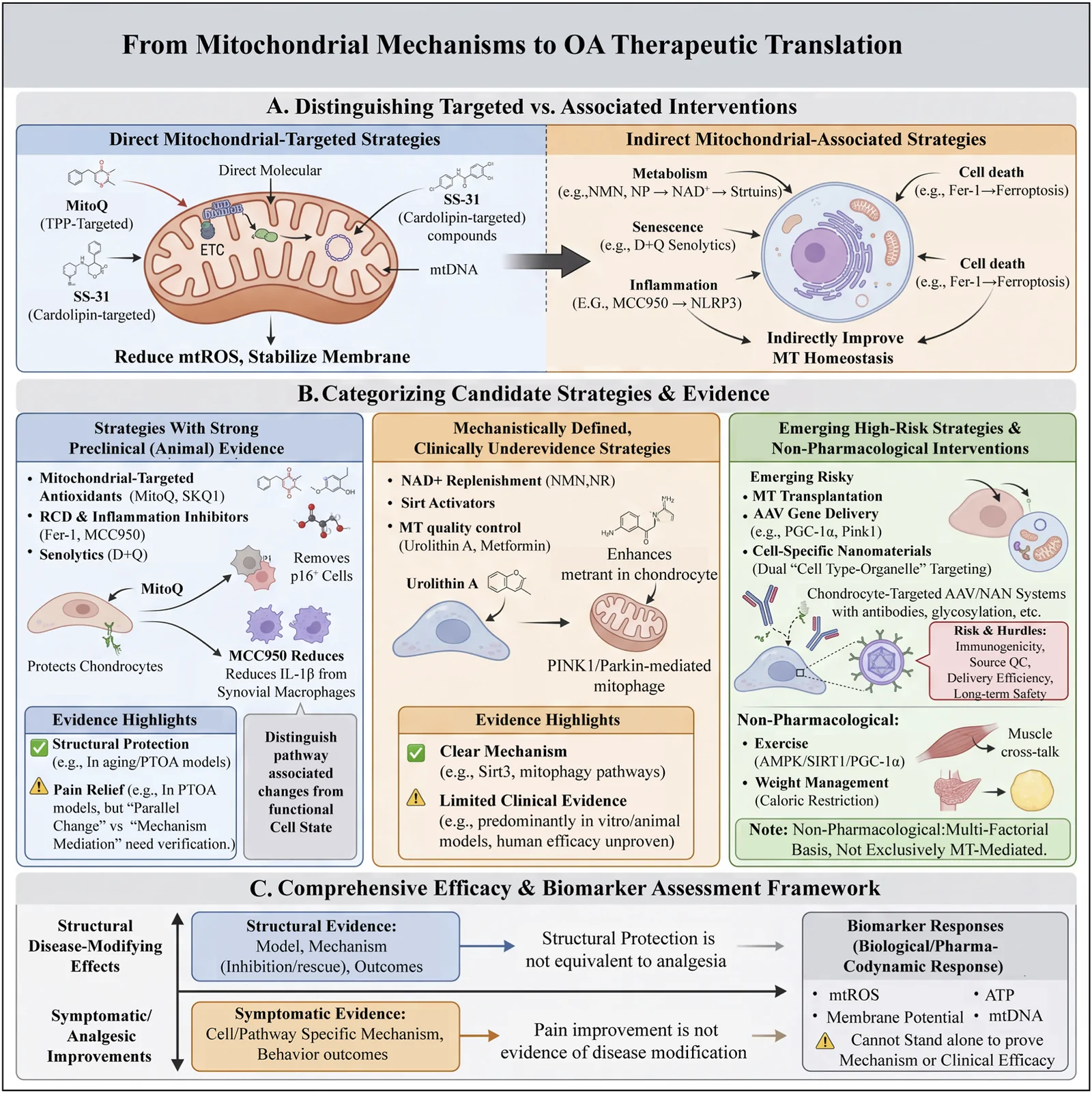
Strategy stratification and comprehensive evaluation framework from mitochondrial mechanisms to osteoarthritis therapeutic translation. This figure outlines the mechanistic classification, levels of evidence, and principles for evaluating efficacy of mitochondria-related therapeutic strategies in OA. **(A)** Direct and indirect mitochondrial interventions: MitoQ, SS-31, and others act directly on mitochondria to reduce mtROS and stabilize mitochondrial membranes, whereas NAD^+^ supplementation, Sirtuin regulation, senolytics, anti-inflammation, and ferroptosis inhibition primarily improve mitochondrial homeostasis indirectly through metabolic, aging, inflammatory, or cell death pathways. **(B)** Strategy selection and evidence stratification: mitochondria-targeted antioxidants, RCD/inflammation inhibitors, and senolytics have relatively strong preclinical evidence; NAD^+^ supplementation, Sirt activation, and mitochondrial quality control strategies have relatively clear mechanisms but limited clinical evidence; mitochondrial transplantation, adeno-associated virus (AAV) gene delivery, cell-specific nanomaterials, and non-pharmacological interventions remain in the exploratory stage. **(C)** Efficacy and biomarker evaluation: structural disease-modifying effects and analgesic benefits should be assessed separately, and pharmacodynamic indicators such as mtROS, ATP, membrane potential, and mtDNA should be incorporated, avoiding direct equating of biomarker changes with mechanistic confirmation or clinical efficacy.

### 7.1 Candidate strategies with relatively strong animal evidence

At present, some mitochondria-related interventions have shown relatively stable cartilage protection, anti-inflammatory, or analgesic effects in OA animal models, but these results mainly reflect preclinical intervention effects and cannot yet be directly extrapolated to clinical efficacy in patients. Mitochondria-targeted antioxidants are one of the more extensively studied classes. MitoQ can accumulate in mitochondria through the triphenylphosphonium (TPP) cation, reduce mtROS, and improve mitochondrial membrane potential (Farshad and Wehrend, 2025). In an aged mouse model of primary OA, MitoQ reduced OA severity and decreased the expression of inflammation- and catabolism-related markers such as MMP-13, iNOS, and NLRP3, mainly supporting its structural protection and anti-inflammatory potential (Hou et al., 2023). SkQ1 in a post-traumatic OA model reduced oxidative stress indicators such as ROS, MDA, and 8-OHdG, and protected mitochondrial membrane potential and mtDNA copy number; related studies also observed improvements in joint function and pain-related behavior (Zhi and Wang, 2024). However, the simultaneous occurrence of improved mitochondria-related indicators and reduced pain behavior does not prove that the former directly mediates the analgesic effect. Direct evidence establishing this mediating relationship through specific intervention, mechanistic blockade, or rescue experiments in pain-related cells or neural pathways is still lacking. Therefore, improvements in mitochondrial function and structural protection, as well as changes in pain behavior, should be interpreted separately as observations at different levels.

Interventions targeting RCD and inflammation-related pathways also have some degree of animal experimental support. Ferrostatin-1 (Fer-1) can scavenge lipid radicals, inhibit lipid peroxidation and ferroptosis-related injury, and maintain GPX4-related antioxidant defense, thereby alleviating IL-1β-induced chondrocyte injury and delaying cartilage degeneration in vivo OA models (Wang X. et al., 2022; Xu W. et al., 2023). These results support a protective effect of Fer-1 against ferroptosis-related injury, but this does not mean that all observed cartilage-protective effects have been proven to be mediated solely by ferroptosis inhibition. The NLRP3 inflammasome inhibitor MCC950 can block inflammasome assembly and caspase-1 activation, reduce IL-1β release, and thereby alleviate synovial inflammation, subchondral bone destruction, and cartilage degeneration (Xin Y. et al., 2022). Therefore, its existing evidence is more appropriately described as inhibition of NLRP3 inflammasome activation and pyroptosis-related signaling, rather than directly equating related molecular changes with the occurrence of complete pyroptosis in all affected cells. In addition, dasatinib plus quercetin (D + Q) can serve as a senolytic strategy targeting senescence-associated cell populations, reduce SASP-related factors, and simultaneously alleviate cartilage degeneration and improve pain behavior in animal models (Gil et al., 2022). Because “senescent cells” are often defined in different studies based on senescence-associated markers such as p16, p21, and SASP, these results are more appropriately interpreted as intervention on senescence-related cell populations and the SASP phenotype, rather than assuming by default that all target cells have been confirmed as irreversibly senescent cells by complete functional criteria. Among these, alleviation of cartilage degeneration belongs to structure-related evidence, whereas improvement in pain behavior belongs to symptom-related evidence, and the two should not substitute for each other as proof of disease-modifying or analgesic effects. Likewise, the parallel occurrence of improvement in SASP or other cellular stress indicators and pain relief cannot alone prove that these molecular changes directly mediate the analgesic effect.

The above strategies have obtained varying degrees of *in vivo* preclinical support, but the strength of their structural disease-modifying evidence and analgesic/symptom evidence is not consistent. For results mainly obtained based on molecular markers, experimental models, or pharmacological interventions, a distinction should be made between “pathway-associated changes” and cell death or aging mechanisms that have already received direct functional validation. For studies that simultaneously report improvement in mitochondrial function or related molecular indicators and reduction in pain behavior, a further distinction should be made between “parallel changes” and “mechanism-mediated” levels of evidence. At present, evidence is still insufficient to establish the disease-modifying or analgesic effects of these strategies in human OA. Future studies should separately validate structural, symptomatic, and mechanistic outcomes, and further clarify long-term safety, administration windows, and applicability across different tissues and patient phenotypes.

### 7.2 Strategies with clear mechanisms but insufficient clinical evidence

Another class of strategies has a relatively clear mechanistic basis but currently remains largely confined to *in vitro* experiments or early animal studies, with insufficient clinical evidence in OA patients. SS-31 (Elamipretide) is a small-molecule peptide targeting cardiolipin in the inner mitochondrial membrane, which can stabilize inner mitochondrial membrane structure, reduce electron leakage and mtROS generation, and inhibit mitochondrial-dependent apoptosis and NLRP3 inflammasome activation (Delco et al., 2018). Although SS-31 has shown protective effects in various diseases related to mitochondrial dysfunction, its direct evidence in OA remains largely preclinical. Therefore, it is currently more appropriate as a candidate strategy with a clear mechanism rather than a therapeutic regimen with established clinical efficacy evidence.

NAD^+^ supplementation and Sirtuins activation are also promising metabolic regulatory strategies. Decreased NAD^+^ levels in OA chondrocytes can lead to reduced SIRT1 and SIRT3 activity, thereby weakening mitochondrial biogenesis, antioxidant defense, and mitochondrial quality control (He Y. et al., 2020). NAD^+^ precursors such as nicotinamide mononucleotide (NMN) and nicotinamide riboside (NR) can increase NAD^+^ levels, restore SIRT1/SIRT3 activity, and promote mitophagy and anti-aging responses. These agents have shown potential to alleviate chondrocyte senescence and matrix degradation in OA models (Wu X. et al., 2025; Li C. et al., 2026). However, NAD^+^ metabolism is not a mitochondria-specific process, and responses to its regulation may differ across cells and tissue compartments. Therefore, metabolic improvement in models can neither prove that its effects are entirely mediated by mitochondria nor directly infer clinical benefit.

Drugs that promote mitochondrial quality control also fall into the category of strategies with clear mechanisms but insufficient clinical evidence. Urolithin A can enhance PINK1/Parkin-mediated mitophagy, promote clearance of damaged mitochondria, and thereby reduce oxidative stress and chondrocyte injury (D'Amico et al., 2022). Rapamycin, metformin, and some natural compounds can also regulate mitophagy, biogenesis, and antioxidant defense through pathways such as AMPK/mTOR, SIRT1/PGC-1α, or Nrf2 (Caramés et al., 2012; Yang Y. et al., 2026). Because these drugs have multiple non-mitochondrial effects, they are more appropriately classified as mitochondria-related rather than directly mitochondria-directed strategies, and the extent to which their OA benefits depend on mitochondrial mechanisms still needs further clarification. In summary, the mechanistic basis for this class of interventions is relatively sufficient, but human OA studies remain limited. Future research should further clarify mitochondrial abnormalities and differences in therapeutic responses among different cell types such as chondrocytes, synovial macrophages, subchondral bone cells, and sensory neurons, to avoid extrapolating a single mitochondrial intervention to all OA phenotypes.

### 7.3 Emerging but higher-risk strategies

In addition to traditional small molecules and metabolic regulators, emerging technologies such as mitochondrial transplantation, AAV gene delivery, and cell-specific nanocarriers provide new possibilities for mitochondria-related interventions in OA, but their translational risk is relatively high (Zhong et al., 2025). Mitochondrial transplantation aims to improve energy metabolism and redox homeostasis in damaged cells through exogenous supplementation of functionally intact mitochondria (Wang et al., 2015). Animal studies have suggested that mitochondrial transplantation can improve energy metabolism in OA chondrocytes, reduce inflammatory responses, and alleviate pain behavior (Lee et al., 2022). However, even if this intervention acts directly on mitochondria, the simultaneous occurrence of improved mitochondrial function and pain relief alone does not permit the conclusion that mitochondrial restoration directly mediates the analgesic effect. Pain improvement may still be attributable to structural protection, reduced inflammation, or other indirect mechanisms, unless verified through specific intervention, blockade, or rescue experiments in pain-related cells or neural pathways. In addition, mitochondrial source, quality control, cell uptake efficiency, immunogenicity, standardized preparation, and long-term safety remain major barriers to its clinical translation.

Gene delivery strategies can regulate mitochondria-protective molecules such as SIRT3, LONP1, PGC-1α, or PINK1/Parkin through AAV or other vectors, thereby achieving relatively long-term regulation of specific mitochondrial pathways (Wang XJ. et al., 2022). Their potential advantage lies in relatively clear targets and sustained effects, but OA has characteristics such as chronicity, heterogeneity, and multi-tissue involvement, and single-gene regulation may not address the diverse pathological compartments. At the same time, the controllability and safety of long-term gene expression still limit their clinical application, so they are currently more suitable as tools for early mechanistic validation and target exploration.

Cell-specific nano-delivery systems may improve the tissue and cell selectivity of mitochondria-related interventions. Although traditional TPP-modified molecules can promote drug enrichment in mitochondria, they lack cell-type specificity and may simultaneously act on chondrocytes, synovial cells, macrophages, and other non-target cells (Han et al., 2019). In the future, antibodies, peptides, glycosylation modifications, or other ligands can be combined to further direct drugs to chondrocytes, synovial macrophages, subchondral bone cells, or sensory nerve-related cells; cartilage-targeted hydrogels or nanoparticles can also prolong intra-articular retention time (Huang et al., 2024). This dual selectivity of “cell type-organelle” has potential advantages, especially for mitochondrial abnormalities dominated by different tissue compartments, but its actual therapeutic benefit still needs to be verified in more rigorous preclinical and clinical studies.

### 7.4 Lifestyle and non-pharmacological metabolic interventions

Lifestyle interventions have advantages such as high safety, long-term feasibility, and obvious systemic metabolic benefits, and are foundational strategies that cannot be ignored in OA management. Exercise training can act by improving muscle function, reducing mechanical load, regulating systemic metabolism, and influencing the local joint microenvironment (Sattler et al., 2023). From the mitochondrial perspective, moderate exercise can activate the AMPK/SIRT1/PGC-1α pathway, promote mitochondrial biogenesis and mitophagy, enhance antioxidant defense, and affect joint tissue metabolism through myokines (Dowden et al., 2023). However, the clinical benefits of exercise have a multifactorial basis, and at present its symptom-improving effects should not be attributed solely to mitochondrial regulation. At the same time, the intensity of exercise should be managed to prevent excessive joint loading, because abnormal mechanical stress may aggravate chondrocyte mitochondrial injury and senescence.

Weight management and caloric restriction can also indirectly protect mitochondrial function by reducing joint mechanical burden and improving metabolic inflammatory status. Obesity-related lipotoxicity can impair chondrocyte mitochondrial homeostasis, promote mtROS generation, inflammatory cytokine release, and cartilage matrix degradation (Vázquez-Mosquera et al., 2021). Through caloric restriction, optimization of dietary structure, and weight control, insulin resistance can be improved, chronic low-grade inflammation reduced, and mitochondrial homeostasis possibly enhanced by increasing NAD^+^ levels and activating SIRT1-related pathways (Podh and Singh, 2026). Similarly, the clinical effects of such interventions involve multiple mechanisms such as mechanical load, systemic metabolism, and inflammation, and mitochondrial changes are more likely to be one component rather than the sole mediator.

Novel metabolic interventions such as hypoxic training simulation and cold exposure have attracted attention in metabolic diseases, but their direct evidence in OA remains limited. Hypoxia-related signaling may modulate HIF-1α-mediated metabolic adaptation of chondrocytes, while cold exposure may influence inflammatory status through systemic energy metabolism and mitochondrial uncoupling (Kang et al., 2020). However, these strategies currently lack sufficient joint-specific studies, and different intensities and durations may produce different or even opposite biological effects. Therefore, in OA they are more suitable as future exploration directions rather than being overemphasized as mature intervention strategies.

In summary, mitochondria-directed and mitochondria-related interventions provide important clues for OA mechanistic research and therapeutic exploration, but therapeutic response itself cannot be regarded as proof of mitochondrial causality. Existing evidence is still mainly based on *in vitro* and animal studies, with limited true human OA clinical evidence, and these strategies exhibit marked differences in their direct mitochondrial effects, structural protection, analgesic efficacy, and cell/tissue specificity. Future research should further clarify mitochondrial abnormalities in different OA phenotypes and tissue compartments, develop cell-specific delivery, and separately evaluate structural disease-modifying and symptom/analgesic outcomes. Therefore, at this stage, it is more appropriate to regard these strategies as candidate interventions with translational potential rather than mature regimens that can already transform OA treatment from “symptomatic treatment” to “mechanism-oriented treatment.” A more accurate judgment of their clinical value will require stratification of mechanistic, animal intervention, and human clinical evidence, combined with mitochondria-related biomarkers and independent structural and symptomatic endpoint evaluation (Table 1).

**TABLE 1:** Mechanistic attributes, structural and analgesic evidence, and translational maturity of mitochondria-targeted and mitochondria-related intervention strategies in OA.

Representative strategy	Target/mechanistic attribute	Main mechanism	Evidence for structural disease modification	Analgesic/symptom evidence	Evidence maturity	Main limitations and translational issues	References
MitoQ, SkQ1	Direct mitochondrial targeting	Accumulate within mitochondria, reduce mtROS, and improve membrane potential and redox homeostasis	Animal models show reduced cartilage damage and decreased catabolic and inflammatory markers; mainly supporting preclinical structural protection potential	Studies on SkQ1 have demonstrated improved joint function and pain-related behavior; independent analgesic evidence for MitoQ is relatively limited	Animal preclinical	Lack of human OA efficacy evidence; relationship between structural and analgesic effects unclear; lack of cell-type selectivity	Farshad and Wehrend (2025), Hou et al. (2023), Zhi and Wang (2024)
Ferrostatin-1 (Fer-1)	Intervention in mitochondria-related cell death pathways	Inhibits lipid peroxidation and ferroptosis	*In vitro* and animal studies support chondrocyte protection and attenuation of cartilage degeneration	OA-specific independent analgesic evidence not yet established	*In vitro*/animal preclinical	Fer-1 is not a direct mitochondria-targeted drug; structural protection cannot be extrapolated to analgesic benefit	Wang X. et al. (2022), Xu W.et al. (2023)
MCC950	Intervention in mitochondria-related inflammatory pathways	Inhibits NLRP3 inflammasome and caspase-1 activation	Animal studies show reduced synovitis, subchondral bone damage, and cartilage degeneration	Analgesic effects lack sufficient independent validation	Animal preclinical	NLRP3 is a downstream inflammatory pathway rather than a direct mitochondrial target; long-term immunological safety needs evaluation	Xin Y.et al. (2022)
Dasatinib + Quercetin (D + Q)	Mitochondria-related senescence intervention	Clears senescent cells and reduces SASP output	Animal studies show reduced cartilage degeneration	Some animal studies observed improved pain behavior	Animal preclinical	Structural and pain effects need independent interpretation; multi-target effects make mitochondria-specific causal attribution difficult	Gil et al. (2022)
SS-31 (Elamipretide)	Direct mitochondrial targeting	Interacts with cardiolipin in the inner mitochondrial membrane, stabilizing the membrane and reducing electron leakage and mtROS.	Direct structural protection evidence in OA remains primarily early preclinical	OA-specific analgesic evidence insufficient	*In vitro*/early animal	Lack of adequate human OA studies; local delivery, dosing, and long-term safety need to be defined	Delco et al. (2018)
NMN, NR, and Sirtuin modulation	Mitochondria-related metabolic intervention	Modulates NAD^+^/SIRT1/SIRT3 network, affecting antioxidant defense, mitochondrial biogenesis, and quality control	Preclinical studies suggest attenuation of chondrocyte senescence and matrix degradation	Limited independent analgesic evidence	*In vitro*/animal preclinical	NAD^+^ metabolism is not mitochondria-specific; significant tissue and cell-type differences exist; human OA efficacy not established	Wu X.et al. (2025), Li C. et al. (2026)
Urolithin A, Rapamycin, Metformin, etc.	Mitochondrial quality control-related/indirect intervention	Regulates mitophagy, biogenesis, and antioxidant responses via PINK1/Parkin, AMPK/mTOR, SIRT1/PGC-1α, or Nrf2	Preclinical studies support chondroprotection and improved cellular homeostasis	Most studies did not independently evaluate analgesic outcomes	*In vitro*/animal preclinical	These agents exhibit multiple non-mitochondrial effects; therapeutic benefits cannot be entirely attributed to mitochondrial modulation	D'Amico et al. (2022), Caramés et al. (2012), Yang Y. et al. (2026)
Mitochondrial transplantation	Direct organelle intervention	Exogenous supplementation of functional mitochondria to improve energy and redox homeostasis	Animal studies suggest improved chondrocyte metabolism, inflammation, and some structural parameters	Some models observed improved pain-related behavior	Proof-of-concept/animal preclinical	It cannot be inferred that mitochondria are the primary driver of OA from combined therapeutic effects; source, uptake efficiency, immunogenicity, quality control, and long-term safety remain unresolved	Wang et al. (2015), Lee et al. (2022)
AAV-mediated SIRT3, LONP1, PGC-1α, PINK1/Parkin modulation	Gene delivery platform; mitochondrial relevance depends on the delivered target.	Long-term modulation of specific mitochondrial or mitochondria-related pathways	Primarily mechanistic and early animal evidence; structural disease modification not yet validated in humans	Insufficient independent analgesic evidence	Early preclinical	AAV itself is not a mitochondrial target; cell specificity, long-term expression risks, and off-target effects need to be defined	Wang X. J. et al. (2022)
Cell-specific nano-delivery/cartilage-targeting materials	This delivery platform enables dual targeting of both cells and organelles	Improves local retention and drug enrichment in specific cells	Mainly supports preclinical cartilage or local tissue protection	Pain improvement evidence depends on the specific payload; currently overall insufficient	Materials science/animal preclinical	Patients with OA may exhibit distinct mitochondrial phenotypes dominated by specific tissue compartments; the *in vivo* distribution, degradation, immunological safety, and scalable production require validation	Han et al. (2019), Huang et al. (2024)
Exercise, weight management, and dietary/caloric intervention	Indirect, systemic mitochondria-related intervention	Affects AMPK/SIRT1/PGC-1α and systemic metabolism, mechanical loading, and inflammatory states	May improve joint loading and metabolic environment, but structural effects cannot be solely attributed to mitochondrial modulation	Although exercise and weight management improve symptoms and function, they do not provide proof of mitochondria-mediated analgesic mechanisms	Human application exists, but mitochondrial mechanism evidence is mainly indirect	Since multiple mechanisms act concurrently, and given significant issues with compliance and individual variability, isolating mitochondria-specific contributions is difficult.	Sattler et al. (2023), Dowden et al. (2023), Vázquez-Mosquera et al. (2021), Podh and Singh (2026)
Hypoxic training mimetics, cold exposure*etc.*	Exploratory metabolic/mitochondria-related intervention.	May affect HIF-1α, energy metabolism, and mitochondrial adaptation	Limited direct structural evidence in OA.	OA-specific analgesic evidence insufficient	Exploratory stage	Dose, duration, and tissue effects uncertain; lack of joint specificity and clinical validation	Kang et al. (2020)

## 8 Translational challenges and future research agenda

Existing studies suggest that mitochondrial abnormalities have been implicated in OA structural pathology, cellular senescence, regulated cell death, and pain-related processes; however, their causal role in human OA, cell specificity, and temporal relationships among different pathological dimensions remain insufficiently clarified. Therefore, future research should prioritize three key objectives: establishing biomarkers that can reflect mitochondrial status and are clinically measurable; clarifying the cellular sources, temporal sequence, and independent contributions of pain-related mitochondrial abnormalities; and developing clinical trial endpoints that can separately evaluate mechanistic effects, symptom improvement, and structural benefits. At the same time, integration of human longitudinal cohorts with joint tissue, body fluid samples, organoids, and joint-on-a-chip platforms should be strengthened to validate the reproducibility and clinical significance of preclinical findings.

### 8.1 Exploration of mitochondrial biomarkers and candidate mechanistic phenotypes

At present, stratification of OA patients still mainly relies on imaging and clinical symptoms, and reliable markers that can dynamically reflect mitochondrial functional status are still lacking. Future research should shift from cross-sectional associations of candidate molecules to analytical validation, longitudinal validation, and clinical utility assessment. Candidate markers can be obtained from multiple sources, including body fluids, tissues, and functional imaging. At the body fluid level, free mtDNA, mtDNA copy number, NAD^+^/NADH ratio, TCA cycle metabolites, ATP levels, and oxidative stress-related indicators in synovial fluid, serum, or urine could be prioritized for evaluation. At the tissue level, single-cell RNA sequencing, spatial transcriptomics, and metabolomics can be integrated to characterize mitochondrial abnormalities in chondrocytes, synovial fibroblasts, macrophages, subchondral bone cells, and sensory nerve-related cells. Further integration with spatial metabolomics, mitochondrial functional imaging, and cell tracing could clarify which cells undergo what mitochondrial changes at different OA stages, and in what tissue microenvironment these changes occur. BOLD-MRI, magnetic resonance spectroscopy, and mitochondrial respiratory chain complex I-related PET tracer strategies can also serve as exploratory tools for non-invasive assessment of local oxygenation and mitochondrial metabolic status.

Prospective studies should simultaneously collect body fluid, tissue, or imaging indicators and combine them with pain phenotypes and structural progression to determine whether specific mitochondrial indicators have predictive value. It is necessary to distinguish state/pharmacodynamic biomarkers from therapeutic efficacy endpoints. Changes in indicators such as mtDNA, NAD^+^/NADH, ATP, or oxidative stress after treatment may reflect target engagement or biological response, but without validation they cannot serve as surrogate endpoints for structural or symptomatic benefit, nor can they alone prove that the mechanism they represent mediates the therapeutic effect. Therefore, biomarker response should not be equated with therapeutic efficacy or a validated surrogate endpoint.

On this basis, hypothetical candidate mechanistic phenotypes such as oxidative stress-dominant, mitochondrial quality control impairment, energy metabolism deficiency, and mitochondrial-pain sensitization-related phenotypes can be explored. These phenotypes currently represent only testable research hypotheses, not established or validated OA clinical subtypes; their stability, reproducibility, and value for patient stratification still need to be validated in independent longitudinal cohorts and treatment response studies.

### 8.2 Longitudinal and cell-specific validation of pain-related mitochondrial abnormalities

Pain sensitization is an important mechanism that underlies the discrepancy between OA structural damage and symptom severity. Future research should move beyond merely demonstrating that “mitochondrial dysfunction is associated with pain” to instead delineate when mitochondrial abnormalities appear in different pain-related compartments, in which cells they occur, and whether they have independent causal roles. In particular, it is necessary to distinguish whether structural lesions and pain sensitization are jointly mediated by shared mitochondrial abnormalities or are separately driven by compartment-specific mitochondrial abnormalities in joint tissues and the nervous system.

Preclinical studies can simultaneously assess joint structure, pain behavior, and mitochondrial function in joint tissues, DRG, and the spinal dorsal horn at early, progressive, and chronic pain stages of OA. These assessments should encompass respiratory chain activity, membrane potential, mtROS, mtDNA, and mitophagy status, and integrate these metrics with mechanical pain threshold, thermal pain threshold, dynamic weight bearing, and gait analysis to determine the temporal relationship between mitochondrial abnormalities and pain sensitization. Furthermore, sensory neuron- or microglia-specific genetic or functional interventions offer a means to validate the roles of key regulatory targets, including SIRT3, PINK1/Parkin, and TFAM, in OA pain. At the same time, tissue/cell-specific interventions targeting chondrocytes, synovial cells, subchondral bone cells, and sensory nerve-related cells should be combined to compare the functional consequences of the same mitochondrial pathway in different compartments, thereby determining whether structural OA progression and pain sensitization depend on shared mitochondrial mechanisms or are mediated relatively independently by mitochondrial abnormalities in different compartments.

Clinical studies can add quantitative sensory testing (QST) to traditional pain intensity scores to evaluate mechanical and thermal pain thresholds, temporal summation, and conditioned pain modulation, and conduct longitudinal combined analyses with mtDNA, NAD^+^/NADH, ATP, and oxidative stress indicators. The candidate “mitochondria-pain” mechanistic phenotypes identified in this way should be considered merely exploratory and hypothesis-generating research frameworks, and whether they can predict persistent pain, identify structural-symptomatic decoupling, or support patient stratification remains to be rigorously validated.

### 8.3 Composite “mechanism-symptom-structure” endpoints for mitochondria-targeted therapy

Clinical trial endpoint design is an important link in the translation of mitochondria-targeted therapies. Traditional OA trials often use joint space width, Kellgren–Lawrence grading, or other structural imaging indicators to evaluate disease-modifying effects, but structural changes are usually slow and are not fully synchronized with pain and functional improvement. For mitochondria-targeted interventions, early effects may first manifest as reduced oxidative stress, improved metabolic status, reduced inflammation, or alleviation of pain sensitization, while obvious structural changes may require longer observation (Zhang et al., 2026c). Therefore, a single structural endpoint may not fully reflect the biological and clinical effects of mitochondria-targeted interventions.

In the future, a three-tier “mechanism-symptom-structure” evaluation framework could be adopted. Within the mechanistic tier, mechanism validation/target engagement should be further distinguished from biomarker or pharmacodynamic response. The former requires evidence from target-specific intervention, mechanism blockade, or rescue to support a causal role; the latter may include mtDNA, NAD^+^/NADH, ATP, oxidative stress, and mitochondrial quality control-related indicators, mainly used to reflect biological or pharmacodynamic changes after treatment. The symptom tier may include visual analogue scale (VAS), WOMAC, patient-reported outcomes (PROs), analgesic use, and gait indicators, with QST and pain mechanism indicators such as conditioned pain modulation added according to research objectives (Kraus et al., 2015). The structural tier may combine imaging, histology, or other indicators that reflect pathological changes in cartilage, synovium, and subchondral bone. The above mechanism validation, biomarker response, symptom benefit, and structural change should be evaluated as interrelated but non-interchangeable dimensions of evidence.

It should be emphasized that improvement in biomarkers or pharmacodynamic indicators first reflects target engagement or biological response, cannot automatically be equated with proof that the mechanism has been demonstrated, and cannot be interpreted as a mediating mechanism of symptom improvement. To further prove that a certain mitochondrial change mediates analgesic effects, it is still necessary to establish a clear temporal sequence, specific interventions in pain-related cells or neural pathways, mechanism blockade or rescue experiments, and, when appropriate, causal mediation analysis. At the same time, structural disease-modifying effects and analgesic/symptomatic benefits should be pre-defined and analyzed as mutually independent but complementary efficacy dimensions, and improvement in either dimension should not be used to infer benefit in the other. This framework helps distinguish different treatment response patterns, such as changes in mechanisms or biomarkers without clear clinical benefit, predominantly symptomatic improvement, predominantly structural protection, or simultaneous benefit across multiple dimensions.

In the future, it is still necessary to further clarify the weighting of each component endpoint, the minimal clinically important difference, the appropriate follow-up time window, and statistical integration methods, and to validate their sensitivity and predictive value through prospective studies. In translational research, mechanism validation, biomarker response, symptom benefit, and structural change should be evaluated separately, and mechanism-mediated interpretation should be made only when corresponding causal evidence is available. Incorporating mechanistic indicators, pain phenotypes, patient-reported outcomes, and structural progression into a unified evaluation system, while separately judging structural disease modification and analgesic effects, may help more accurately evaluate the clinical translational value and potential applicable populations of mitochondria-related interventions (Table 2).

**TABLE 2:** Priority research directions for translating mitochondrial biology into precision management of osteoarthritis.

Future research direction	Core question	Suggested methods	Key indicators	ranslational value
Mitochondrial biomarkers	Can they reliably reflect mitochondrial status and have value for disease stratification or outcome prediction?	Prospective longitudinal cohorts, validation across multiple body fluids and tissues, and analytical and clinical validity evaluation	mtDNA, NAD^+^/NADH, ATP, oxidative stress indicators	Patient stratification, biological/pharmacodynamic response monitoring; after validation, explore their value for disease progression or therapeutic efficacy prediction
Cell and spatial typing	Which cells and tissue regions dominate mitochondrial abnormalities?	Single-cell sequencing, spatial omics, metabolomics	Cell-specific mitochondrial expression and metabolic features	Target identification, mechanistic typing
Mitochondria-pain phenotype	Which patients have pain sensitization associated with mitochondrial abnormalities?	Longitudinal pain assessment, QST, DRG/spinal cord mechanism validation	mtROS, mtDNA, ATP, QST, VAS/WOMAC.	Identification of candidate pain mechanism phenotypes and patient stratification; the predictive value of these phenotypes requires further validation
Mechanism-symptom-structure composite endpoints	How to distinguish mechanism validation, biomarker response, and structural/symptom efficacy, and comprehensively evaluate mitochondria-related interventions?	Prospective clinical trials, multidimensional endpoint integration, combined with stratified analysis of target engagement, pharmacodynamic indicators, and clinical outcomes	Mechanism/target engagement indicators + mitochondrial biomarkers + WOMAC/QST + structural indicators such as qMRI.	Distinguish mechanism proof, biological response, and therapeutic efficacy, optimize efficacy evaluation and clinical trial design

## 9 Conclusion

A growing body of evidence indicates that mitochondrial dysfunction is an important contributing factor to OA-related metabolic stress, cell aging, regulated cell death, and inflammatory reaction, and may also participate in pain sensitization. Impaired respiratory chain function, mtROS accumulation, mitochondrial dynamics imbalance, insufficient mitophagy, and mtDNA damage can disrupt cellular energy metabolism and redox homeostasis, and are associated with SASP release, matrix degradation, inflammation amplification, and abnormal cell fate. Existing evidence provides relatively sufficient support for the roles of these mechanisms in cartilage and other joint tissues; however, the independent causal contribution of mitochondrial abnormalities to the occurrence and sensitization of OA pain remains inadequately validated. Therefore, it cannot currently be inferred that the same mitochondrial pathological process is simultaneously responsible for OA structural progression and pain sensitization merely because similar mitochondrial abnormalities exist in structurally related tissues and pain-related neural compartments.

From the perspective of the whole joint and nervous system, mitochondrial abnormalities can occur in cartilage, synovium, subchondral bone, immune cells, sensory neurons, and the central neuro-glial system. These changes may occur relatively independently, or may form bidirectional or multidirectional feedback through mtROS, mtDNA/DAMPs, inflammatory mediators, and neuroimmune signaling. Therefore, at this stage, it is more appropriate to regard mitochondrial dysfunction as a shared metabolic-inflammatory pathological node affecting multiple joint and neural compartments, rather than as an already established single mechanistic bridge linking cartilage degeneration to pain sensitization. Structural pathology, local inflammation, peripheral sensitization, and central sensitization may influence each other, but their temporal sequence, cellular sources, and causal direction have not yet been fully clarified.

On this basis, the “metabolism-senescence-pain axis” proposed in this paper is more suitable as a hypothesis-generating integrative framework for summarizing the possible links among metabolic abnormalities, cell aging, and pain-related processes, and should not be regarded as a proven continuous mechanistic pathway. Similarly, oxidative stress dominance, impaired mitochondrial quality control, energy metabolism defects, and pain sensitization-related features can currently only be explored as hypothetical candidate mechanistic phenotypes, rather than as established OA molecular or clinical subtypes. Future research should focus on validating these emerging hypotheses, including clarifying the temporal relationships and causal contributions of mitochondrial abnormalities across different tissues and cellular compartments through human longitudinal studies, establishing mitochondrial-related biomarkers with analytical and clinical validity, and evaluating the reproducibility and patient stratification value of candidate mechanistic phenotypes. At the same time, constructing composite “mechanism-symptom-structure” endpoints that can separately evaluate mechanistic effects, symptom improvement, and structural benefits will help more accurately distinguish established pathological mechanisms from cross-compartment links that still require validation. Future translational research should also clearly distinguish mechanistic proof, biomarker response, and therapeutic efficacy, avoiding the direct equating of changes in mitochondrial indicators with causal mechanisms or clinical benefits. On this basis, stratified evaluation of mechanistic validation, biomarker/pharmacodynamic response, symptom benefit, and structural changes may help more accurately determine the actual value of mitochondrial-related mechanisms and intervention strategies in OA research and clinical translation.
